# Exploring the Role of *HNRNPA3* in Breast Cancer Progression, Immune Microenvironment, and Therapeutic Sensitivity: A Multiomics and Functional Prediction Study

**DOI:** 10.1155/humu/5519745

**Published:** 2026-05-03

**Authors:** Lijie Gong, Yang Xu, Weihui Guo, Xufan Cai, Fanrong Zhang, Jie Ma, Houquan Tao, Weiliang Feng

**Affiliations:** ^1^ Department of Breast Surgery, Zhejiang Cancer Hospital, Hangzhou, Zhejiang, China, zchospital.com; ^2^ Hangzhou Institute of Medicine (HIM), Chinese Academy of Sciences, Hangzhou, Zhejiang, China, cas.cn; ^3^ Department of Thoracic Surgery, Zhejiang Cancer Hospital, Hangzhou, Zhejiang, China, zchospital.com; ^4^ Key Laboratory of Gastroenterology of Zhejiang Province, Zhejiang Provincial People′s Hospital, Affiliated People′s Hospital, Hangzhou Medical College, Hangzhou, Zhejiang, China, hznu.edu.cn; ^5^ School of Basic Medical Sciences and Forensic Medicine, Hangzhou Medical College, Hangzhou, Zhejiang, China, hznu.edu.cn; ^6^ Cancer Center, Department of Thoracic Surgery, Zhejiang Provincial People′s Hospital, Affiliated People′s Hospital, Hangzhou Medical College, Hangzhou, Zhejiang, China, hznu.edu.cn; ^7^ Department of Pathology, Zhejiang Provincial People′s Hospital, The Affiliated People′s Hospital of Hangzhou Medical College, Hangzhou, Zhejiang, China; ^8^ General Surgery, Cancer Center, Department of Gastrointestinal and Pancreatic Surgery, Zhejiang Provincial People′s Hospital, The Affiliated People′s Hospital of Hangzhou Medical College, Hangzhou, Zhejiang, China

**Keywords:** breast invasive carcinoma (BRCA), drug sensitivity, *HNRNPA3*, immune infiltration, single-cell RNA sequencing (scRNA-seq), spatial transcriptomics, therapeutic target, tumor microenvironment (TME)

## Abstract

**Background:**

Breast invasive carcinoma (BRCA) remains a leading cause of female cancer mortality, necessitating novel biomarkers and therapeutic targets. Heterogeneous nuclear ribonucleoprotein A3 (HNRNPA3) emerges as a potential regulator in tumor progression and immune modulation, yet its comprehensive role in BRCA remains uncharacterized.

**Methods:**

We conducted an integrated multiomics analysis of HNRNPA3 in BRCA using data from TCGA, GEO datasets, single‐cell RNA sequencing, and spatial transcriptomics. Bioinformatics approaches included differential expression analysis, survival analysis, functional enrichment, immune microenvironment characterization, and drug sensitivity prediction.

**Results:**

HNRNPA3 was significantly upregulated in BRCA tissues and correlated with advanced tumor grade, metastasis, and poor prognosis across multiple cohorts. Functional enrichment revealed HNRNPA3′s involvement in cell cycle regulation and immune‐related pathways. Immune profiling demonstrated that high HNRNPA3 expression was associated with altered immune cell distribution, particularly CD4+ T cells, and reduced immunotherapy response. Spatial transcriptomics confirmed predominant HNRNPA3 expression in malignant regions. Drug sensitivity analysis identified potential therapeutic agents (CD‐437 and talazoparib) targeting HNRNPA3‐associated pathways.

**Conclusion:**

HNRNPA3 functions as a critical oncogenic regulator in BRCA by promoting tumor progression through cell cycle dysregulation and immune microenvironment remodeling. Its strong association with therapy resistance positions HNRNPA3 as both a prognostic biomarker and promising therapeutic target for breast cancer intervention strategies.

## 1. Introduction

Breast invasive carcinoma (BRCA), as the most prevalent malignancy among women worldwide, also represents the leading cause of cancer‐related mortality in females [1]. The urgency to understand its etiology, develop effective treatments, and implement preventive strategies is underscored by the high incidence and mortality rates associated with breast cancer [2, 3]. Therefore, there is a critical demand for identifying and creating novel, effective biomarkers and targeted treatments.

The tumor microenvironment (TME) of breast cancer is a complex and dynamic ecosystem that critically influences disease progression, therapeutic response, and clinical outcomes. Characterized by significant heterogeneity across molecular subtypes, the breast cancer TME exhibits distinct immune cell compositions, ranging from immunologically “hot” tumors with robust T‐cell infiltration to “cold” tumors dominated by immunosuppressive elements such as regulatory T cells (Tregs), tumor‐associated macrophages (TAMs), and myeloid‐derived suppressor cells (MDSCs) [2, 4]. T cells play a pivotal role in antitumor immunity through three primary mechanisms: direct tumor cell cytotoxicity, immune response modulation, and immunological memory formation [5, 6]. However, within the TME, T cells frequently exhibit an exhausted phenotype characterized by functional impairment and upregulated expression of inhibitory receptors (e.g., PD‐1 and CTLA‐4). Furthermore, the TME presents substantial barriers to T cell infiltration, including physical obstacles such as a dense extracellular matrix and chemical barriers comprising immunosuppressive molecules [7, 8]. Notably, tumors with limited T cell infiltration typically demonstrate poor responsiveness to immunotherapeutic interventions [9, 10]. Hence, it is of great importance to gain a deeper understanding of the role and mechanism of immunological regulation in the microenvironment of BRCA and search for immune‐related genes and proteins.

As part of the hnRNP family, *HNRNPA3* contributes to essential biological processes, including cell cycle modulation, cellular differentiation, checkpoint control, and responses to DNA damage [11, 12]. Its altered expression in certain cancers, such as liver, glioma, and colorectal, suggests a potential involvement in tumor development. During the development of hepatocellular carcinoma (HCC), *HNRNPA3* levels rose consistently across all stages, with higher expression linked to reduced survival rates. For example, *HNRNPA3* expression has been found to increase gradually in the progression from cirrhosis to HCC, and its expression level can be used to differentiate between high‐grade dysplastic nodule (HGDN) and early hepatocellular carcinoma (eHCC) [13]. In bladder cancer, elevated *HNRNPA3* expression was significantly associated with lymph node metastasis and demonstrated a strong correlation with increased risks of disease progression and cancer‐specific mortality [14]. The protein levels of *HNRNPA3* exhibit a significant increase in T‐47D, Colo16, HeLa, A549, and RPMEI cell lines as compared with normal keratinocytes [15].

However, a detailed and systematic investigation into the pathological implications and the intricate molecular underpinnings of BRCA, particularly regarding the role of *HNRNPA3* in immune microenvironment regulation, immune scoring, and drug sensitivity, remains insufficient. This highlights a significant gap in the current understanding of the disease′s progression and therapeutic targets. In this study, we have addressed this gap by comprehensively exploring *HNRNPA3*′s involvement in immune modulation, immune infiltration patterns, and its potential as a predictive biomarker for drug response, thereby providing new insights into its therapeutic relevance in BRCA.

## 2. Materials and Methods

### 2.1. Acquisition of Genomic Sequencing Datasets

To explore the potential biological mechanisms and clinical prognostic significance of *HNRNPA3*, we obtained multiple datasets, including normal tissue expression data (TPM, Transcripts Per Kilobase of exon model per Million mapped reads) from GTEx via UCSC (https://genome.ucsc.edu/), tumor expression data (TPM) from TCGA, breast cancer expression matrices and clinical information from GEO datasets (GSE3494, GSE9893, GSE97394, GSE22219, and GSE21653), single‐cell RNA‐seq datasets (E‐MTAB‐8107 and GSE176078 from GEO), and spatial transcriptomics data (GSM6433587 and GSM6433588).

### 2.2. Receiver Operating Characteristic (ROC) Curve Analysis

We used cancer (*n* = 1088) and normal tissue (*n* = 288) samples from the TCGA‐GTEx dataset. The values were normalized using z‐score scores, and if the z‐score was greater than 3.0 or less than −3.0, the value was classified as an outlier and removed. Subsequently, we calculated the 95% confidence intervals and the area under the curve (AUC) for *HNRNPA3* using the pROC package [16].

### 2.3. Survival Analysis

KmPlot (https://www.kmplot.com/) is a free online platform for plotting survival curves, which includes data on breast cancer, colorectal cancer, and gastric cancer, among others [17]. We used this website to calculate and visualize the prognostic level of HNRNPA3 in breast cancer and its different molecular subtypes.

### 2.4. Genomic Alteration Analysis

Genomic alteration profiles of *HNRNPA3* across 33 cancer types were analyzed using somatic mutation and copy number variation data from The Cancer Genome Atlas (TCGA) Pan‐Cancer Atlas cohort (*n* = 6051 samples), downloaded from the Genomic Data Commons (GDC) Data Portal. Mutation annotation format (MAF) files were processed with the *maftools* R package to compute gene‐wise alteration frequencies, identify recurrent hotspot mutations, and generate lollipop plots integrated with protein domain annotations (RRM_10 domain).

### 2.5. Immune Infiltration and Immunotherapy Prediction

Using the median expression of *HNRNPA3* from TCGA‐BRCA, GSE9893, GSE97394, GSE22219, and GSE21653 datasets as the cutoff, samples were divided into high‐ and low‐expression groups. Seven immune infiltration algorithms (CIBERSORT, TIMER, xCell, MCPcounter, ESTIMATE, EPIC, and quanTIseq) were applied, and the results were sorted by *HNRNPA3* expression levels from low to high. Cell types with significant expression differences were screened using the Wilcoxon test, and the correlation coefficients and their significance with *HNRNPA3* expression levels were calculated.

TIP (tracking tumor immunophenotype) is a dedicated server for analyzing tumor immunophenotypes. It swiftly processes data and visually assesses the distribution of tumor‐infiltrating immune cells across seven stages of the cancer immune cycle: cancer cell antigen release (Stage 1), cancer antigen presentation (Stage 2), immune cell priming and activation (Stage 3), immune cell migration to the tumor (Stage 4), immune cell infiltration into the tumor (Stage 5), T cell recognition of cancer cells (Stage 6), and cancer cell killing (Stage 7) [18]. Following submission of TCGA‐BRCA data to the TIP portal (http://biocc.hrbmu.edu.cn/TIP/), Spearman′s rank correlation was utilized to determine potential correlations between computed TIP scores and *HNRNPA3* expression profiles.

Chemokine‐related genes, immunostimulatory factors, and immunosuppressive markers were obtained from TISIDB (http://cis.hku.hk/TISIDB/download.php; [19]). The dataset was stratified into high‐ and low‐expression subgroups according to *HNRNPA3* expression levels. Differential expression analysis was conducted using the Wilcoxon rank‐sum test, with results presented in a heat map visualization.

TIDE is a computational framework and database designed to predict patient responses to immune checkpoint blockade (ICB) therapy [9, 20]. It achieves this by analyzing mechanisms of T cell dysfunction and T cell exclusion within the TME. In this study, we utilized the TIDE platform (http://tide.dfci.harvard.edu/) to analyze TCGA‐BRCA data. We assessed the relationships between *HNRNPA3* and key immunotherapy‐related metrics, including Responder, TIDE score, IFNG, Merck18, CD274, CD8, Dysfunction, Exclusion, MDSC, and CAF. *HNRNPA3* expression levels were stratified into quartiles (Q1, Q2, Q3, and Q4) based on cutoffs of 0.75, 0.5, and 0.25 for visualization. Additionally, we downloaded 25 publicly available immunotherapy datasets from TIDE. By integrating *HNRNPA3* expression levels with immunotherapy outcomes, we calculated the AUC values and visualized the results.

### 2.6. Drug Sensitivity Prediction

In this study, we utilized three comprehensive databases: the Genomics of Drug Sensitivity in Cancer (GDSC), Cancer Therapeutics Response Portal 2.0 (CTRP2.0), and the PRISM Lab. GDSC Version 1 encompassed 987 cell lines and 367 compounds, whereas Version 2 included 809 cell lines and 198 compounds [21]. We employed Spearman′s rank correlation to determine the correlation coefficients and *p* values between gene expression levels and IC50 (half‐maximal inhibitory concentration) values. Within the GDSC cgp2016 dataset, we utilized the “pRRophetic” tool to calculate IC50 values and their correlations for samples within the TCGA‐BRCA cohort.

The CTRP2.0 and PRISM databases provided the area under the dose‐response curve (AUC) values for various cell lines, which are integral for assessing drug efficacy across different cellular contexts. Through integration of transcriptomic data from the Cancer Cell Line Encyclopedia (CCLE), we systematically analyzed associations between gene expression patterns and pharmacological response profiles.

In the pursuit of novel therapeutics, we have harnessed the power of the eXtreme Sum (XSum) algorithm to navigate the expansive repository of small molecules within the Connectivity Map (CMAP) database. This sophisticated computational approach evaluates the therapeutic potential of these molecules, with the guiding hypothesis that a compound′s lower score on the XSum scale is indicative of its superior therapeutic value.

### 2.7. Functional Gene Set Enrichment

We defined the top 30% of samples with the highest *HNRNPA3* expression as the high‐expression group, and the bottom 30% with the lowest expression as the low‐expression group. We performed differential analysis between the two groups using the limma function [22]. The differentially expressed genes were sorted by LogFC. We then used the clusterProfiler package [23] to conduct Gene Ontology (GO) analysis based on the GO‐BP (biological process), GO‐MF (molecular function), and GO‐CC (cellular component) gene sets, as well as reactome and wikipathways gene sets. Additionally, we executed the Kyoto Encyclopedia of Genes and Genome (KEGG) analysis using the fgsea function in the fgsea package based on the KEGG gene set to obtain the normalized enrichment score (NES). Results with a *p* < 0.05 and an adjusted *p* < 0.25 were considered significant and were visualized.

CancerSEA (Cancer Single Cell Expression Atlas) database is a valuable resource that includes single‐cell expression data of cancer cells across 14 functional states and their characteristic genes (stemness, invasion, metastasis, proliferation, EMT, angiogenesis, apoptosis, cell cycle, differentiation, DNA damage, DNA repair, hypoxia, inflammation, and quiescence) [24]. We utilized the z‐score function in the R package gene set variation analysis (GSVA) [25] to compute combined z‐scores for the 14 functional state gene sets imported from the database. Subsequently, we applied the scale function to the results for further standardization. Finally, we calculated the Pearson correlation between *HNRNPA3* and the scores of each gene set.

### 2.8. Single‐Cell RNA‐Seq Analysis and Spatial Transcriptome Analysis

In this study, we utilized the Sparkle database (https://grswsci.top/) and the SpatialTME platform (https://www.spatialtme.yelab.site/) to perform spatial transcriptomic analysis of BRCA. The Cottrazm package was employed to deconvolute the cellular composition of the TME using the `get_enrichmentget_enrichment_matrix and enrichment_analysis functions, which were critical for generating an enrichment matrix of diverse cell types. The spatial distribution of major cell types within each microregion was visualized using the SpatialFeaturePlot function from the Seurat package, which also enabled the mapping of *HNRNPA3* expression across individual spots. Spots were categorized into three groups based on malignancy scores: “Malignant”(score = 1, indicating the presence of malignant cells), “Normal” (score = 0, indicating the absence of malignant cells), or “Mixed” (scores between 0 and 1, indicating a mixture of cell types). Spearman correlation analysis was applied to assess the relationships between cellular composition and gene expression levels across all spots, with visualizations generated using the linkET package.

Single‐cell data were analyzed using the Seurat package [26]. Genes in < 3 cells and cells with < 200 genes or > 20% mitochondrial content were excluded. Normalization was carried out using the LogNormalize function. To reduce dimensionality and visualize cell clusters, uniform manifold approximation and projection (UMAP) was utilized, projecting the data into a two‐dimensional space. Cluster‐specific marker genes were identified with the “FindAllMarkers” function. The “DotPlot” function in Seurat was used to illustrate the expression profiles of these marker genes across clusters.

### 2.9. Cell Culture and Tissue Samples

Human breast cancer cell lines, including MDA‐MB‐231, MDA‐MB‐468, MCF7, MDA‐MB‐453, and HCC1806, as well as the human normal breast cell line Bcap37, were cultured at the Key Laboratory of Gastroenterology of Zhejiang Provincial People′s Hospital (Zhejiang, China). All cell lines used in this study were obtained from the American Type Culture Collection (ATCC). The cancer cell lines were cultured in either RPMI 1640 or Dulbecco′s Modified Eagle Medium (DMEM) (ServiceBio, Wuhan, China, Cat. G4538 and G4515) supplemented with 10% fetal bovine serum (FBS, Mylscience, Shenyang, China, Cat. M‐FBS01). The cells were maintained in an incubator set to 37°C with a 5% carbon dioxide atmosphere.

### 2.10. Overexpression Plasmid and sh‐*HNRNPA3* Lentivirus

Lentiviral vectors encoding sh‐*HNRNPA3* or empty vector (pCDH), along with *HNRNPA3* overexpression *HNRNPA* (*HNRNPA3*‐OE) or corresponding empty vector (pCDH), were generated by GeneChem (Shanghai, China). These lentiviral constructs were subsequently transduced into breast cancer cell lines MDA‐MB‐231 and MDA‐MB‐453. The target sequence for *HNRNPA3*‐specific shRNA was 5 ^′^‐AGGTGATGGTGGATATAAT‐3 ^′^.

### 2.11. RNA Extraction and Quantitative Real‐Time PCR (qRT‐PCR)

Total RNA was isolated from cells using the FastPure Cell/Tissue Total RNA Isolation Kit V2 (Vazyme Biotech, Nanjing, China). cDNA synthesis was performed using the HiScript RT SuperMix for qPCR (+gDNA wiper) (Vazyme Biotech, Nanjing, China). Quantitative PCR was carried out using SYBR Green, and data were acquired on the Applied Biosystems 7500 Real‐Time PCR System. Relative gene expression levels were calculated using the 2^−*Δ* *Δ*Ct^ method. Primers were designed and synthesized by Repobio (Hangzhou, China), and their sequences are listed below:

*HNRNPA3*‐qF:ACGTTCCAGGGGCTTTGGT; *HNRNPA3*‐qR:TGGTTCCACTACACGCCCA.

### 2.12. TUNEL Staining

We selected the One‐step TUNEL In Situ Apoptosis Kit (Green, FITC, by Elabscience, Cat. E‐CK‐A320) and conducted the experiment in accordance with the provided protocol. Following depolymerization and tissue hydration, the sections were subjected to Protease K for 20 min at 37°C. After balanced by 100 *μ*L 1 × DNase I Buffer, 100 *μ*L DNase I working solution (200 U/mL) was added for 20 min at 37°C. Subsequently, we added 50‐*μ*L marking working solution on the slides and incubated in the dark for 60 min at 37°C. After nucleic acid staining with DAPI, we observed and photographed the samples using the FITC filter on a fluorescence microscope.

### 2.13. Cell Proliferation Assay

To assess cell proliferation, the Cell Counting Kit‐8 (CCK‐8; Elabscience, Wuhan, China, Cat. E‐CK‐A362) was utilized. MDA‐MB‐231 and MDA‐MB‐453 cells were dissociated and seeded into 96‐well culture plates at a density of 2 × 10^3^ cells per well, with five replicate wells for each condition. The plates were then incubated overnight in a humidified incubator at 37°C with 5% CO2. At 24‐, 48‐, and 72‐h postseeding, 10 *μ*L of CCK‐8 reagent was added to each well, followed by a 2‐h incubation period. Absorbance was subsequently measured at 450 nm using a microplate reader. All experiments were independently repeated three times to ensure reproducibility.

### 2.14. Transwell Migration and Invasion Assays

MDA‐MB‐231 and MDA‐MB‐453 cell lines, transfected with either pCDH or *HNRNPA3*, were seeded at a density of 5 × 10^4^ cells per well in serum‐free medium onto the upper compartment of Transwell inserts with 8‐*μ*m pores (Corning, United States). For the migration assay, the inserts were used as received. In contrast, for the invasion assay, the inserts were precoated with Matrigel and incubated at 37°C for 30 min. The lower chamber was filled with 500 *μ*L of DMEM supplemented with 10% FBS. Following a 24‐h incubation period, cells that had migrated or invaded through the pores were fixed with 4% paraformaldehyde and stained with crystal violet. Nonmigrated or noninvaded cells on the upper surface were removed with a cotton swab. The number of cells that had penetrated the membrane was quantified using ImageJ software. The scratch assay was performed to evaluate cell migration capacity. Briefly, cells were seeded in six‐well plates and cultured to 100% confluence. A sterile pipette tip was used to create a uniform wound in the cell monolayer, followed by incubation in serum‐free medium. Wound closure was monitored and imaged at 0, 12, and 24 h using phase‐contrast microscopy.

### 2.15. In Vivo Study

This research adheres to relevant applicable ethical standards. The mouse experiments were carried out in line with the protocols established by the Gastroenterology Key Laboratory of Zhejiang Province. Four‐ to five‐week‐old female BALB/c nude mice, with a body weight ranging from 14 to 16 g, were obtained from the Animal Research Center at Zhejiang Provincial People′s Hospital and kept in a pathogen‐free environment. Each mouse received a subcutaneous injection of MDA‐MB‐453 cells (5 × 106 cells in 200 *μ*L) into the right flank. Tumor volume was calculated using the equation *V* = *L* (*l* *e* *n* *g* *t* *h*) × *W* (*w* *i* *d* *t* *h*)^2^/2. Following the study′s completion, the mice were humanely sacrificed by cervical dislocation, and their tumors were removed, documented, weighed, and collected for further analysis.

### 2.16. Statistical Analysis

Statistical analyses were performed using R software (Version 4.4.1) and GraphPad Prism 9.0. Data are presented as *m* *e* *a* *n* ± *s* *t* *a* *n* *d* *a* *r* *d* *d* *e* *v* *i* *a* *t* *i* *o* *n* (SD). Differences between groups were assessed using Student′s t‐test or one‐way ANOVA, with a *p* *v* *a* *l* *u* *e* < 0.05 considered statistically significant.

## 3. Results

### 3.1. The *HNRNPA3* Expression Status and Clinical Character in Breast Cancer

In pan‐cancer differential expression analysis, we found that *HNRNPA3* is differentially expressed in various cancers, with a relatively higher expression in cancer tissues across most cancer types (Figure 1a). *HNRNPA3* demonstrates high accuracy in distinguishing cancer from normal tissue, with an area under the ROC curve (AUC) of 0.894, indicating a good diagnostic performance. (Figure 1b). In GSE3494, we found that *HNRNPA3* is expressed at higher levels in high‐grade tumor samples, such as the G3 (*p* = 0.0075) and T2‐3 groups (p = 0.00021) (Figure 1c,d). In GSE9893, we observed that high expression of *HNRNPA3* is more prevalent in samples with lymph node metastasis (*p* = 0.0013) and distant metastasis (*p* = 0.00021) (Figures 1f, 1g, 1h, 1i, 1j, and 1k). Subsequently, we discovered that samples with high expression of *HNRNPA3* exhibited poorer prognosis in terms of overall survival (OS) (*p* = 0.033), distant metastasis‐free survival (DMFS) (*p* = 0.0038), progression‐free survival after progression (PPS) (*p* = 1.3*e* − 06), and relapse‐free survival (RFS) (*p* = 3.5*e* − 13) (Figures 1l,m).

Figure 1The expression and significance of HNRNPA3 in TCGA‐GTEx and GEO datasets. (a) The expression of HNRNPA3 in pan‐cancer.  ^∗^ *p* < 0.05,  ^∗∗^ *p* < 0.01, and  ^∗∗∗^ *p* < 0.001. (b) The area under the ROC curve (AUC) of HNRNPA3 in TCGA‐GTEx datasets. (c–e) The clinical significance of HNRNPA3 in GSE3494. (f–i) The clinical significance of HNRNPA3 in GSE9893. (j–m) Survival curve of HNRNPA3 in KmPlot datasets including overall survival (OS), distant metastasis‐free survival (DMFS), progression‐free survival after progression (PPS), and relapse‐free survival (RFS).

**Figure:**
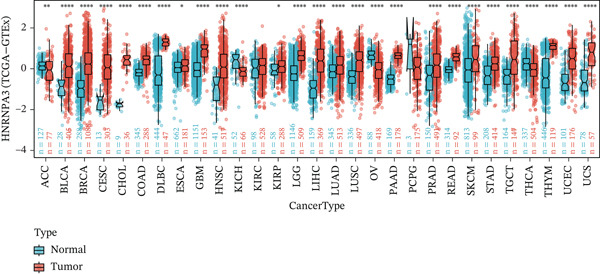
(a)

**Figure:**
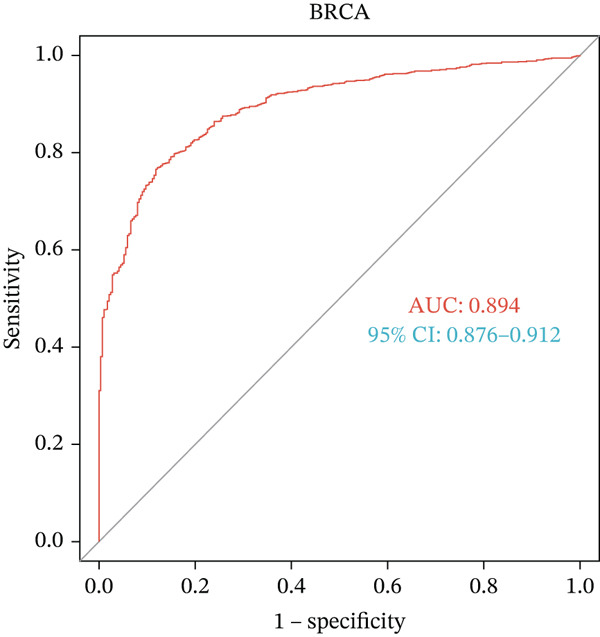
(b)

**Figure:**
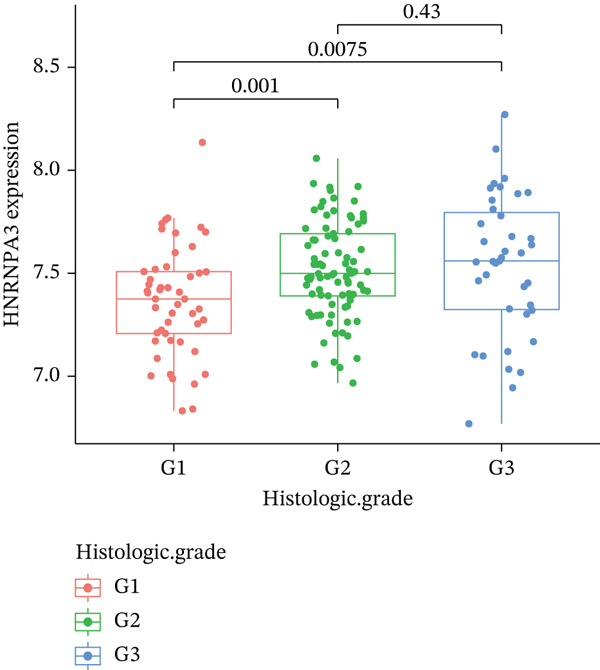
(c)

**Figure:**
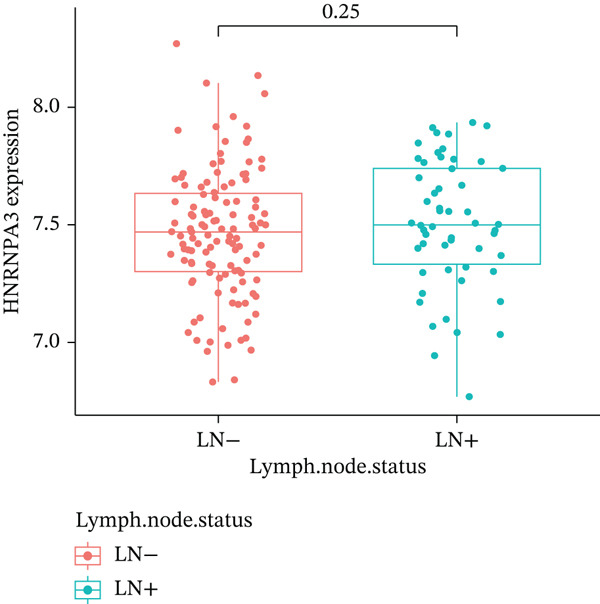
(d)

**Figure:**
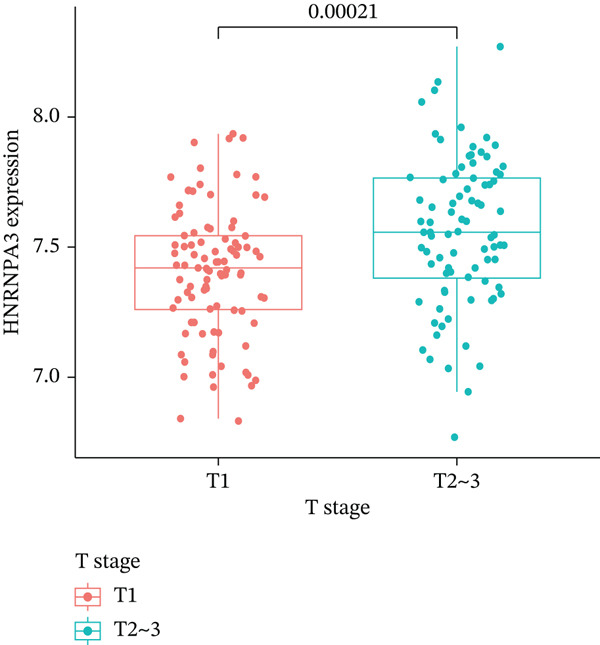
(e)

**Figure:**
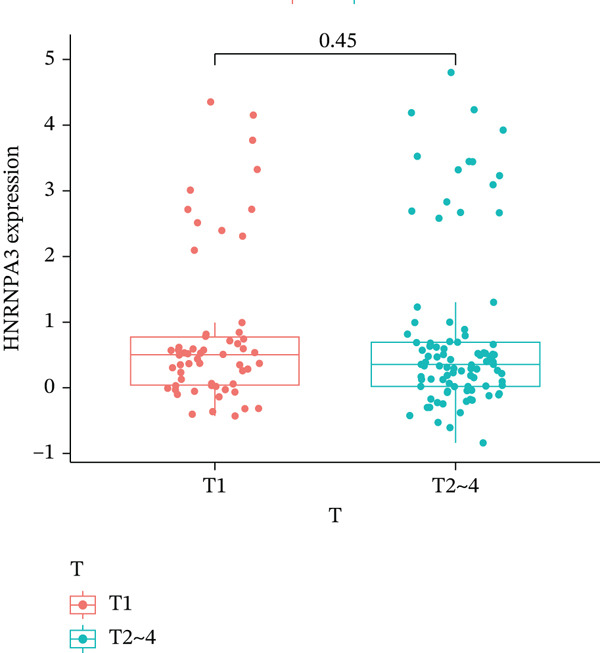
(f)

**Figure:**
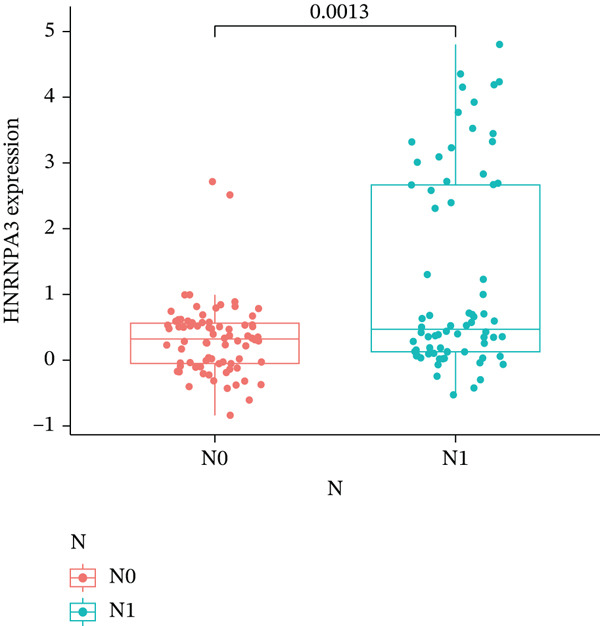
(g)

**Figure:**
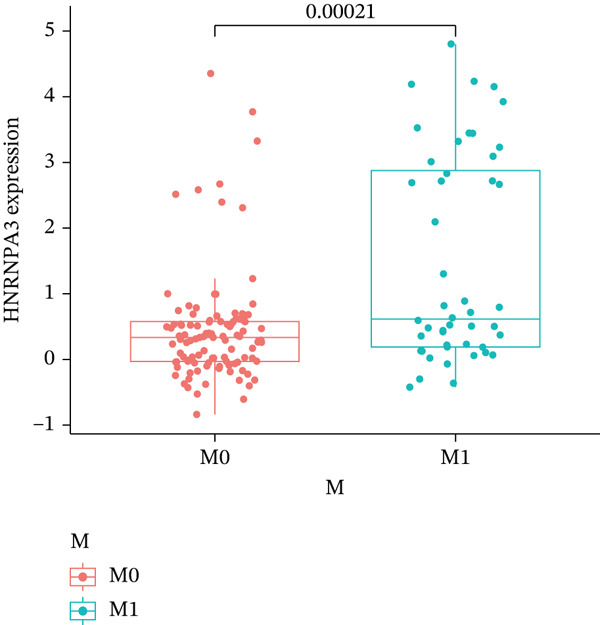
(h)

**Figure:**
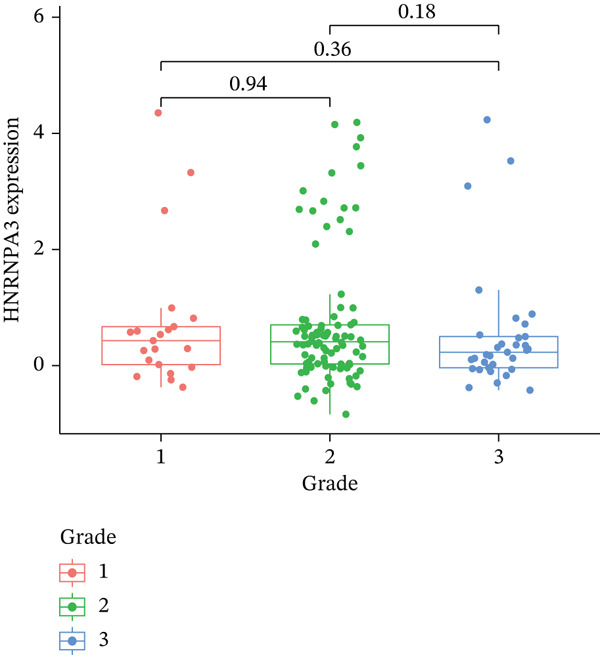
(i)

**Figure:**
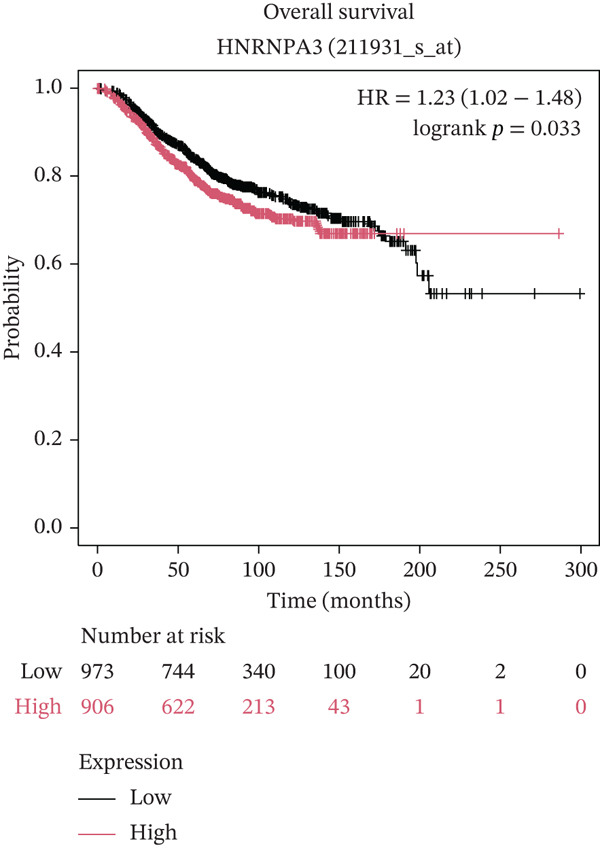
(j)

**Figure:**
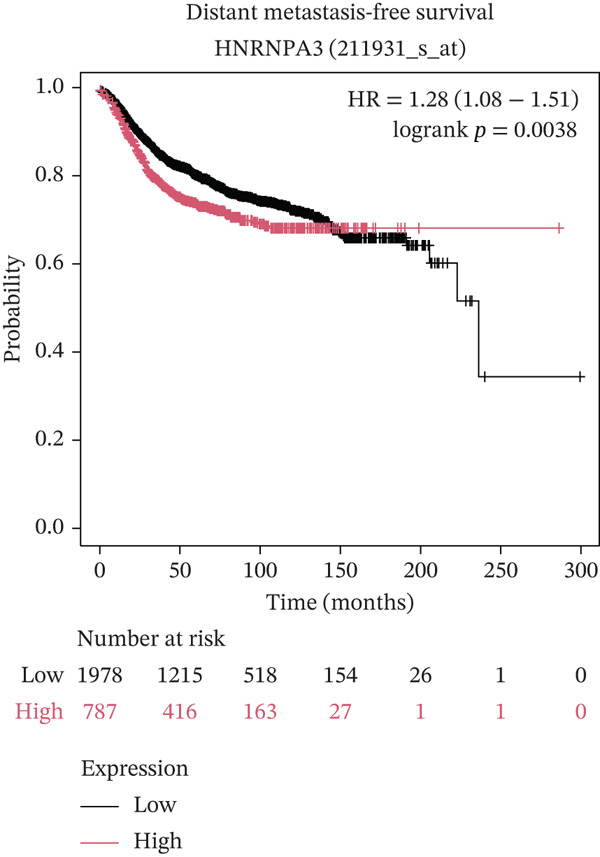
(k)

**Figure:**
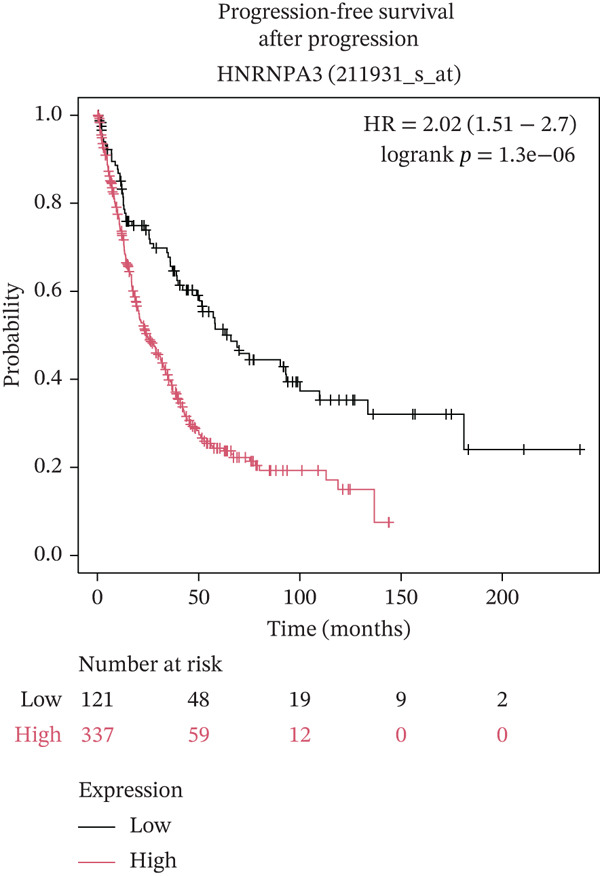
(l)

**Figure:**
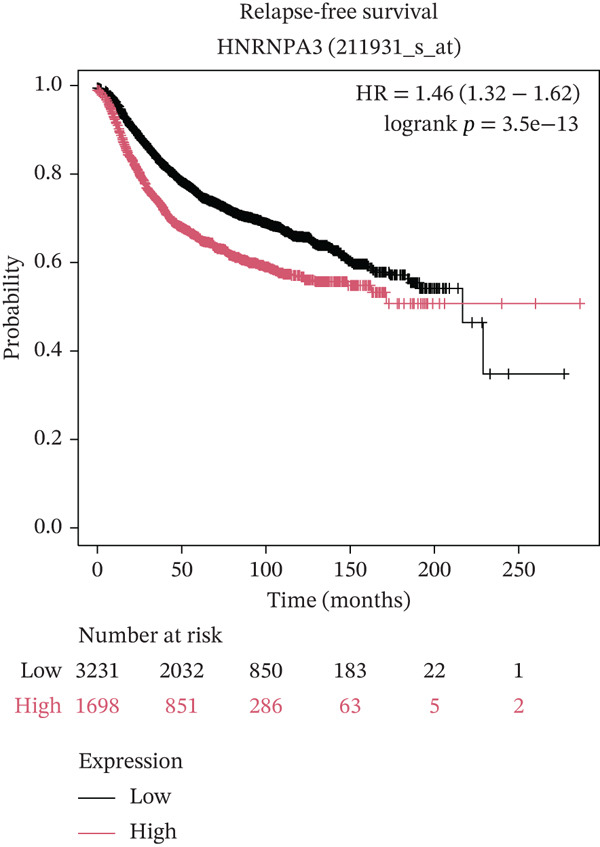
(m)

### 3.2. *HNRNPA3* Functional Enrichment Analysis

In the GO pathway prediction, we focused primarily on five aspects: c2.cp.reactome, c2.cp.wikipathways, c5.go.bp, c5.go.cc, and c5.go.mf, and visualized the top five pathways with the NES values. We found that cell cycle‐related pathways were significantly enriched in the high expression *HNRNPA3* group, such as Wp Cell Cycle (NES = 2.5), Reactome M Phase (NES = 2.4), and Reactome Cell Cycle Checkpoints (NES = 2.7) (Figure 2a). Additionally, we observed that chemokine‐related pathways were also enriched in the high expression *HNRNPA3* group, including Gomf Chemokine Activity (NES = 2.3) and Gomf Chemokine Receptor Binding (NES = 2.2) (Figure 2a).

Figure 2Functional enrichment analysis in breast cancer. (a) GO pathway analysis in TCGA‐BRCA including c2.cp.reactome, c2.cp.wikipathways, c5.go.bp, c5.go.cc, and c5.go.mf. (b) KEGG pathway analysis in TCGA‐BRCA. (c) GSVA analysis in the TCGA‐BRCA dataset. (d) GSVA analysis in the GSE9893 dataset. NES, normalized enrichment scores.

**Figure:**
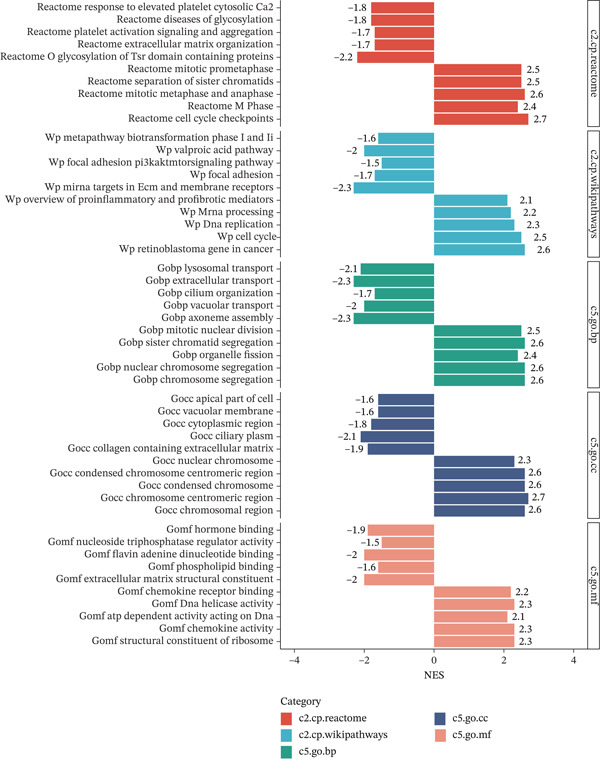
(a)

**Figure:**
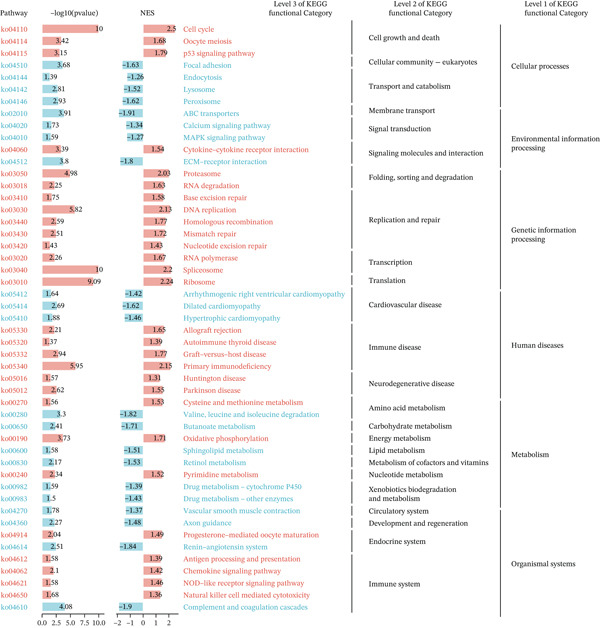
(b)

**Figure:**
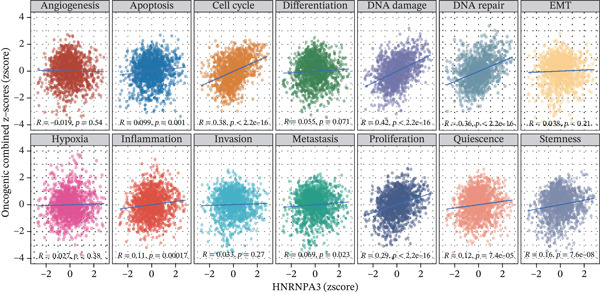
(c)

**Figure:**
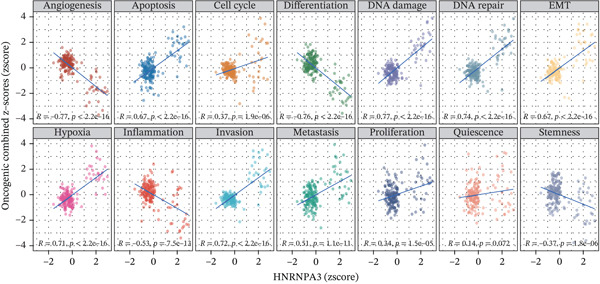
(d)

In the KEGG pathway analysis, we found significant enrichment of the cell cycle (NES = 2.5) and Chemokine signaling pathway (NES = 1.42) in samples with high *HNRNPA3* expression, which is in agreement with the results from the GO analysis. Moreover, we identified substantial enrichment of several Immune system pathways, including antigen processing and presentation (NES = 1.39), NOD‐like receptor signaling pathway (NES = 1.46), and natural killer cell mediated cytotoxicity (NES = 1.36) (Figure 2b).

GSVA surpasses traditional pathway prediction by detecting nuanced changes in pathways between samples. In our study, we analyzed 14 tumor cell functional states in the TCGA‐BRCA and GSE9893 datasets. We found that in TCGA‐BRCA, *HNRNPA3* was significantly correlated with Cell Cycle (R = 0.38, *p* < 2.2*e* − 16), DNA damage (R = 0.42, *p* < 2.2*e* − 16), DNA repair (R = 0.36, *p* < 2.2*e* − 16), and proliferation (R = 0.29, *p* < 2.2*e* − 16) (Figure 2c). In the GSE9893 dataset, *HNRNPA3* was associated with angiogenesis (R = −0.77, *p* < 2.2*e* − 16), apoptosis (R = 0.67, p < 2.2e − 16), cell cycle (R = 0.37, *p* = 1.9*e* − 06), differentiation (R = −0.76, *p* < 2.2*e* − 16), and DNA damage (R = 0.77, *p* < 2.2*e* − 16) (Figure 2d).

### 3.3. *HNRNPA3* in Immune Infiltration and Immunotherapy Outcome Prediction

To comprehensively and systematically analyze the role of *HNRNPA3* in the breast cancer immune microenvironment, we selected seven immune deconvolution algorithms (CIBERSORT, TIMER, xCell, MCPcounter, ESTIMATE, EPIC, and quanTIseq) to evaluate the immune cells associated with *HNRNPA3*. Using heat maps and the Wilcoxon rank‐sum test, we visualized the distribution of significantly altered immune cells in two groups based on median values. We observed differential distributions of various cell types in the heat maps, particularly T cell CD4+ and macrophages (Figure 3a). Subsequently, we conducted immune infiltration analysis across multiple datasets, including TCGA‐BRCA, GSE9893, GSE97394, GSE22219, and GSE21653, and calculated Spearman correlation coefficients with HNRNPA3 expression levels (Figure 3b). Among them, the correlation between T cell CD4 + Th2 and *HNRNPA3* in the TCGA‐BRCA dataset was particularly significant (R = 0.38, *p* < 2.2*e* − 16) (Figure 3c). Compared with the low *HNRNPA3* expression group, the high‐*HNRNPA3* expression group exhibited higher ‐mmune score and ESTIMATE score (*p* < 0.01) (Figure 3d). In the high‐*HNRNPA3* expression group, chemokines, immunostimulators, and immunoinhibitors were commonly overexpressed (Figure 3e). To further elucidate the relationship between *HNRNPA3* and immune regulation, we conducted GSEA analysis. We observed significant enrichment of chemokines (NES = 2.371, *p* < 0.001), immunostimulators (NES = 1.886, *p* < 0.001), and immunoinhibitors (NES = 1.768, *p* = 0.002) in the high‐*HNRNPA3* expression group (Figures 3f, 3g, and 3h). In the TIP score, we found that *HNRNPA3* is predominantly positively correlated with Step 3 (pricing and activation), Step 4 (immune cell recruiting), and Step 7 (killing of cancer cells), whereas it shows a negative correlation with Step 5 (infiltration of immune cells into tumors) (Figure 3i). In the TCGA‐BRCA samples, we used the EaSIeR package to predict chemokine scores and found that the group with high *HNRNPA3* expression had lower chemokine score levels (*p* < 0.001) (Figure 3j).

Figure 3The relationship between *HNRNPA3* and the immune microenvironment. (a) The relationship between *HNRNPA3* and the outcomes of seven immunological algorithms (CIBERSORT, TIMER, xCell, MCPcounter, ESTIMATE, EPIC, and quanTIseq). (b) Heat map depicting the correlation between *HNRNPA3* and various immune cells in TCGA‐BRCA, GSE9893, GSE97394, GSE22219, and GSE21653 datasets. The color of the squares represents the correlation coefficient (*p* < 0.05). Red: closer to 1 (positive correlation); blue: closer to −1 (negative correlation). A cross (×): *p* ≥ 0.05. (c) The correlation between *HNRNPA3* and T cell CD4+ Th2. (d) The StromalScore, Immune score, and ESTIMATE score between the *HNRNPA3* low‐ and high‐expression groups.  ^∗^ *p* < 0.05,  ^∗∗^ *p* < 0.01,  ^∗∗∗^ *p* < 0.001. (e) Differences in gene sets for chemokines, immunostimulators, and immunoinhibitors between high‐ and low‐*HNRNPA3* expression groups. (f–h) GSEA enrichment analysis of gene sets for chemokines, immunostimulators, and immunoinhibitors in high versus low‐*HNRNPA3* expression groups. (i) Correlation plot between the TIP (tracking tumor immunophenotype) score and *HNRNPA3* expression. (j) Differences in chemokine scores between the high‐ and low‐*HNRNPA3* expression groups.

**Figure:**
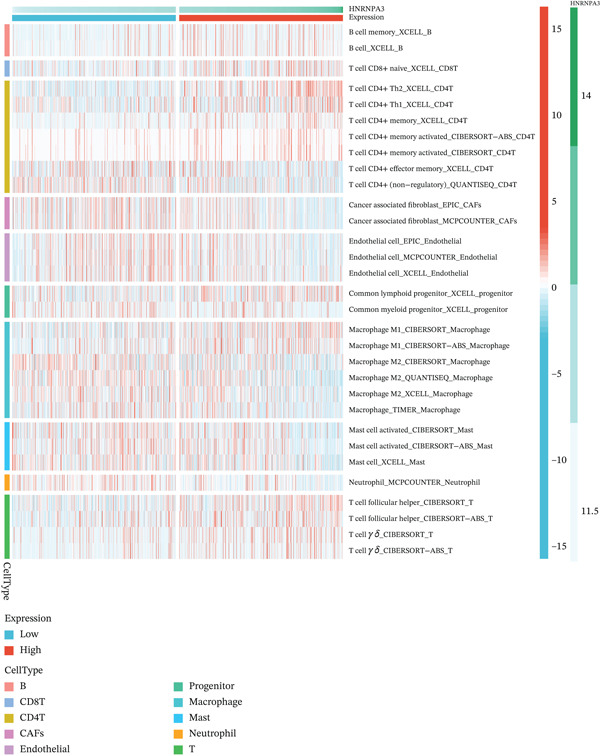
(a)

**Figure:**
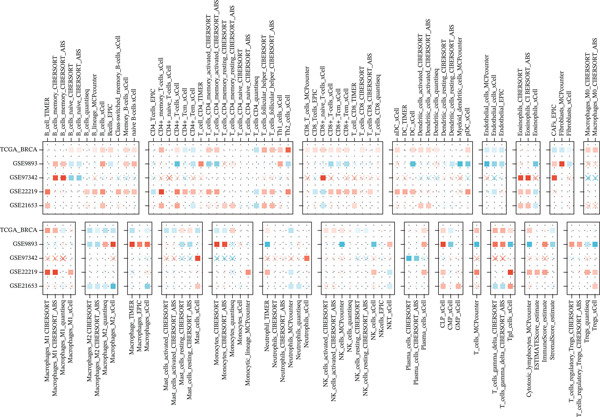
(b)

**Figure:**
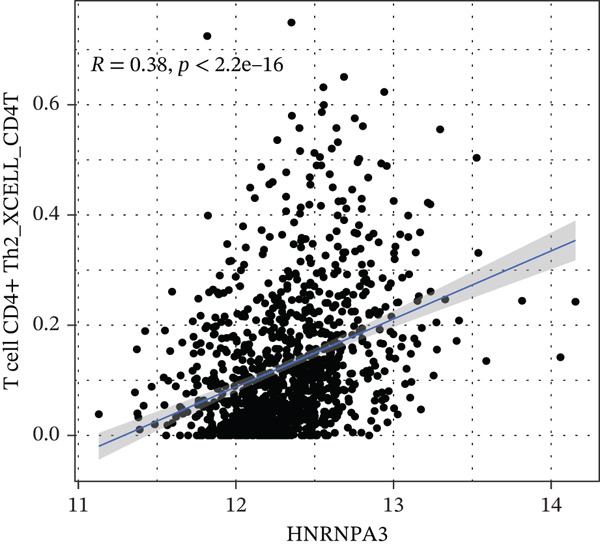
(c)

**Figure:**
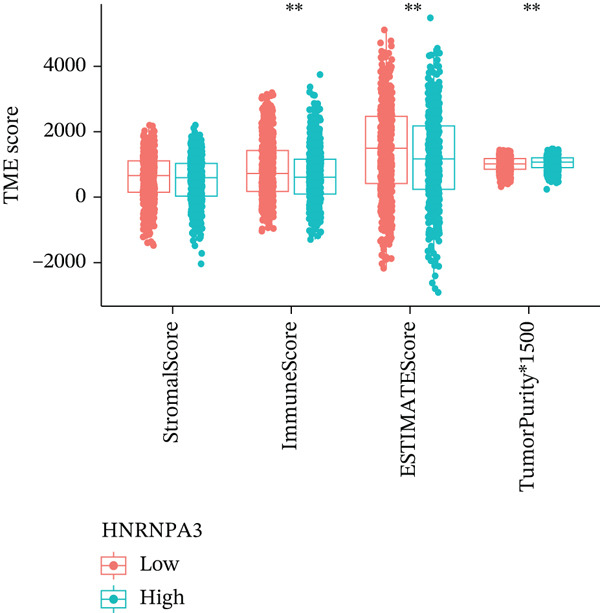
(d)

**Figure:**
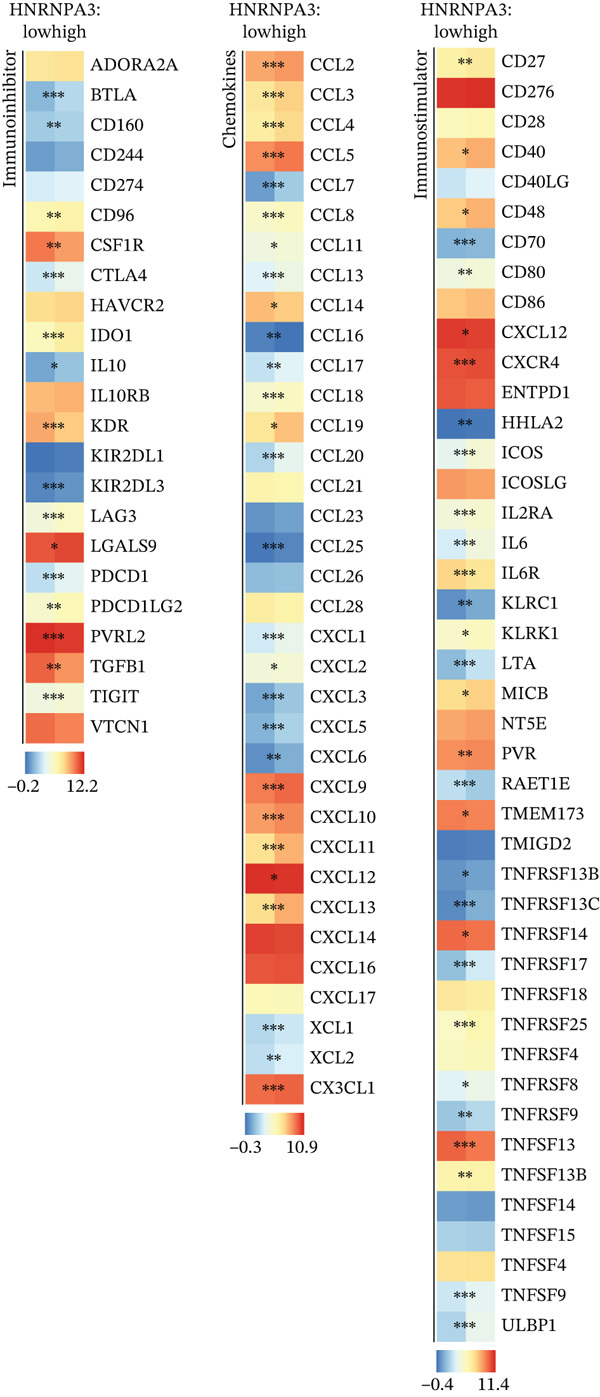
(e)

**Figure:**
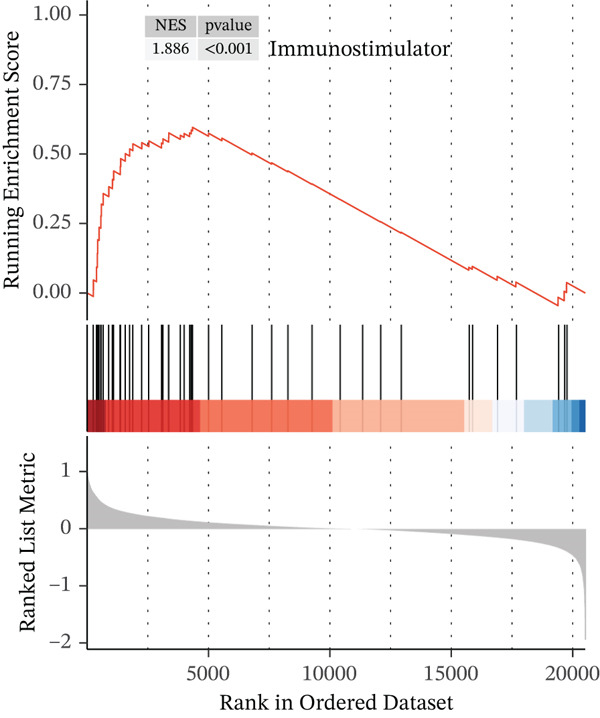
(f)

**Figure:**
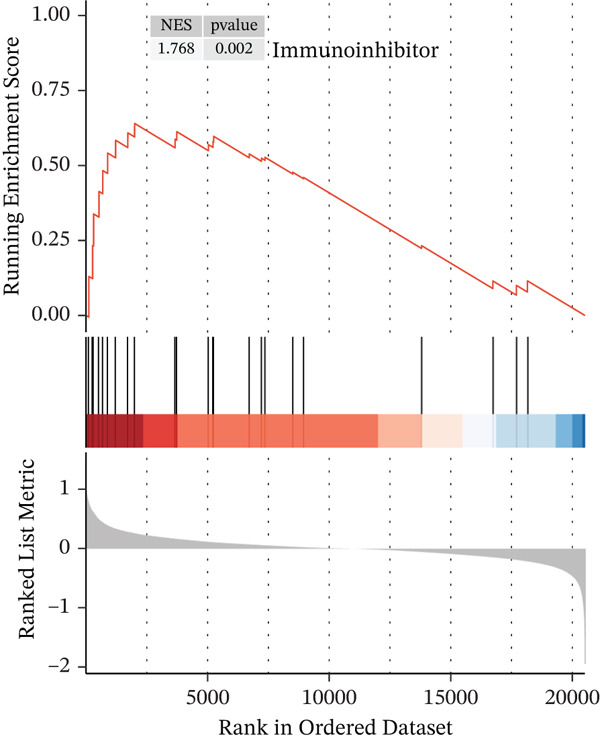
(g)

**Figure:**
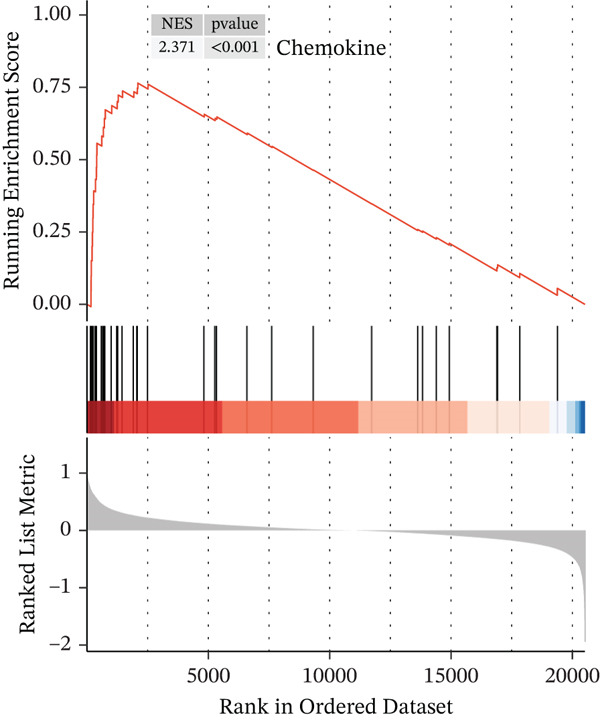
(h)

**Figure:**
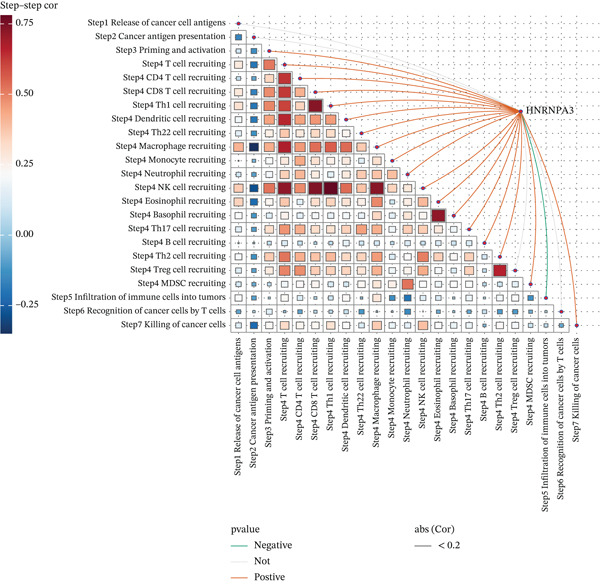
(i)

**Figure:**
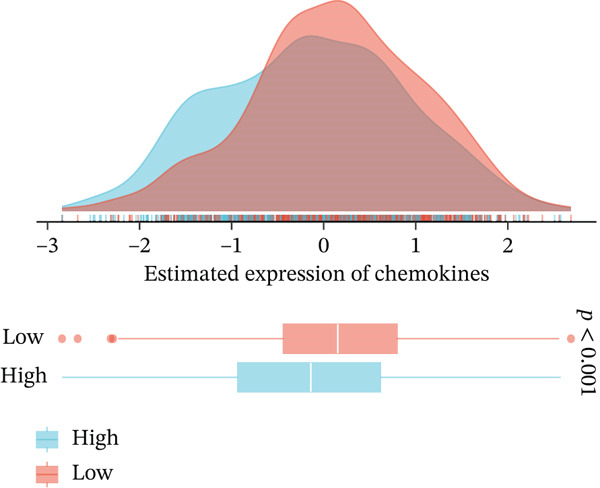
(j)

The above findings suggest that *HNRNPA3* is closely associated with multiple pathways in the immune microenvironment and immune processes. Based on this, we hypothesized that *HNRNPA3* might be linked to immunotherapy outcomes. To test this hypothesis, we utilized the TIDE database (http://tide.dfci.harvard.edu/) for validation. Among the 25 publicly available immunotherapy‐related datasets, *HNRNPA3* demonstrated strong predictive performance in Zhao2019_PD1_Glioblastoma_Pre (AUC = 0.68), Nathanson2017_CTLA4_Melanoma_Pre (AUC = 0.70), and Gide2019_PD1_Melanoma (AUC = 0.73) (Figure 4a). We further employed the response prediction module within TIDE for online analysis. The results revealed that the Q2 (Q2: intermediate *HNRNPA3* expression 25%–50%) and Q3 (Q3: intermediate *HNRNPA3* expression 50%–75%) groups exhibited significantly higher values in TIDE score, IFNG, Merck18, CD274, CD8, MDSC, CAF, and exclusion compared with the Q4 group (lowest HNRNPA3 expression, bottom 25%). In contrast, the Q4 group showed higher values in dysfunction (Figures 4b, 4c, 4d, 4e, 4f, 4g, 4h, 4i, and 4j).

Figure 4Association of *HNRNPA3* with immunotherapy outcomes and immune microenvironment features. (a) Predictive performance of *HNRNPA3* in immunotherapy response across 25 independent datasets. (b–j) Analysis of *HNRNPA3* expression groups (Q1: highest expression, top 25%; Q2: 25%–50%; Q3: 50%–75%; Q4: lowest expression, bottom 25%) using TIDE′s response prediction module. IFNG, interferon gamma; MDSC, myeloid‐derived suppressor cells; CAF, cancer‐associated fibroblasts; TMB, tumor mutational burden; TIDE, tumor immune dysfunction and exclusion.

**Figure:**
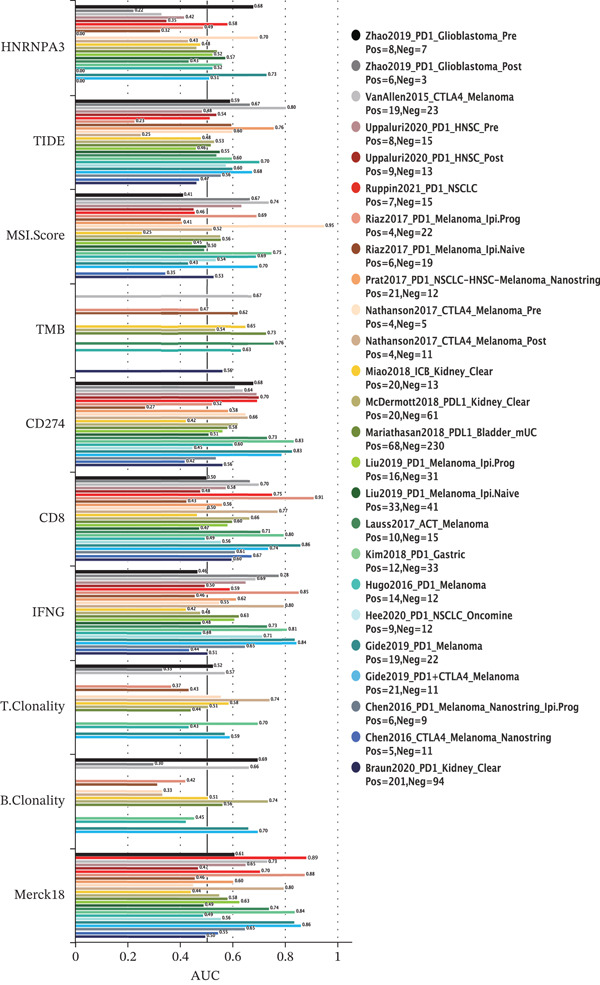
(a)

**Figure:**
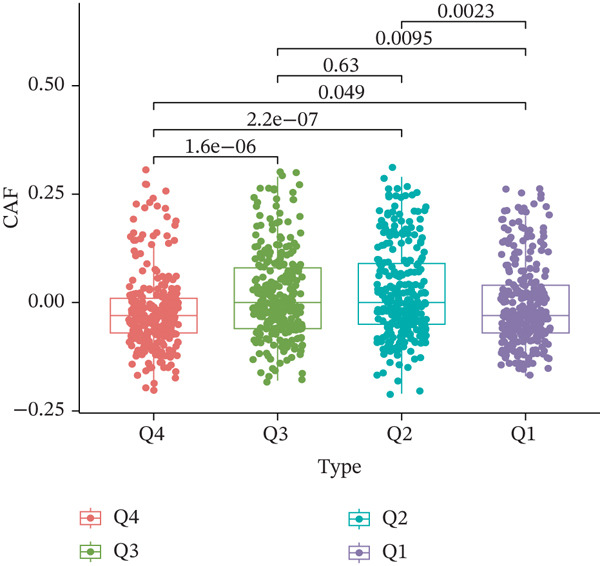
(b)

**Figure:**
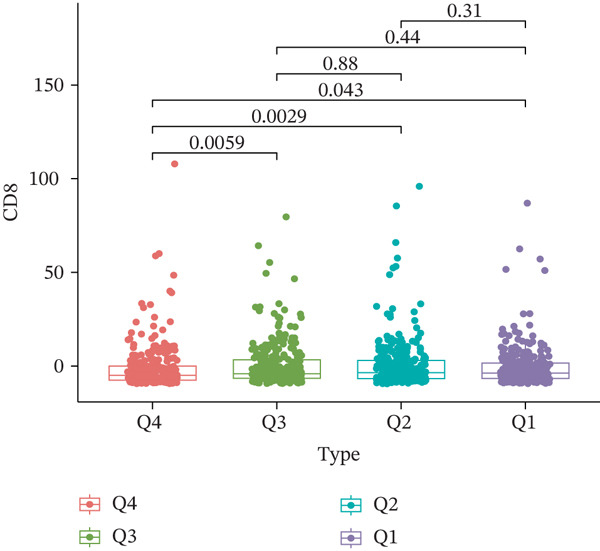
(c)

**Figure:**
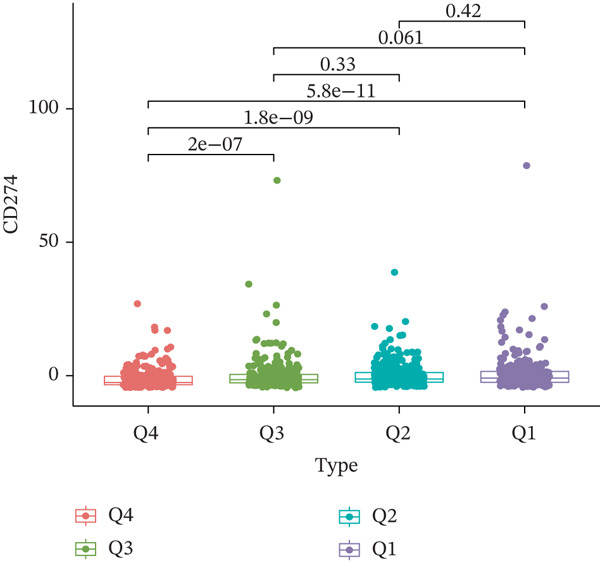
(d)

**Figure:**
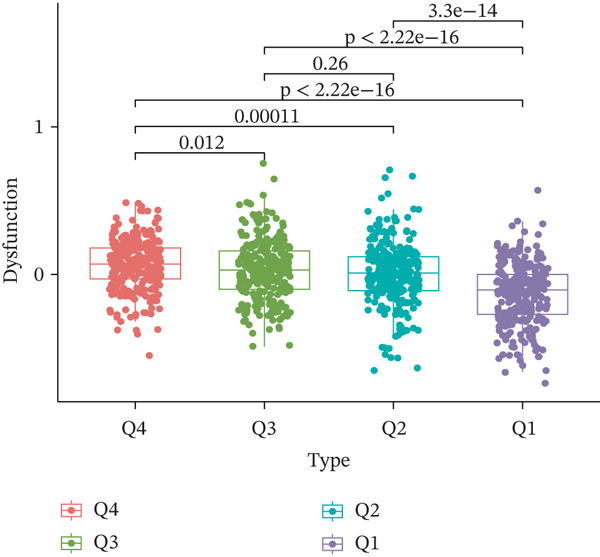
(e)

**Figure:**
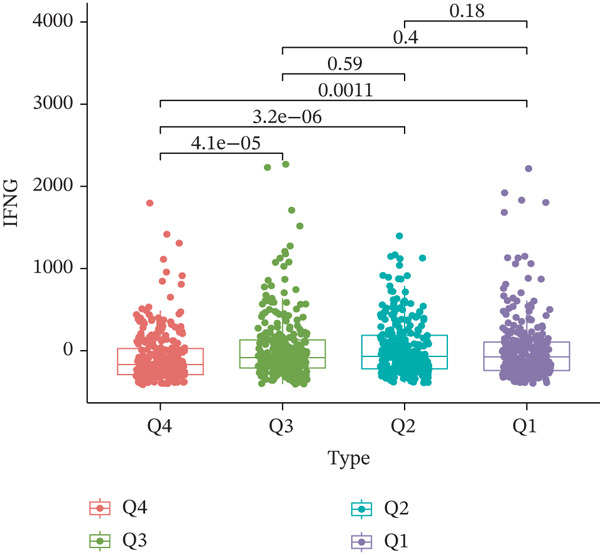
(f)

**Figure:**
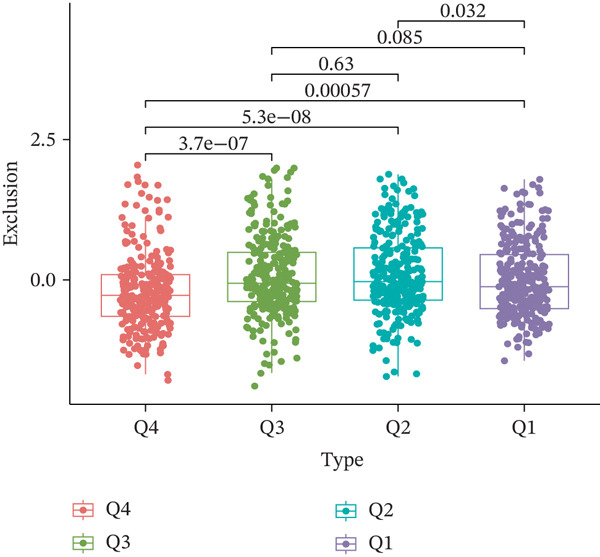
(g)

**Figure:**
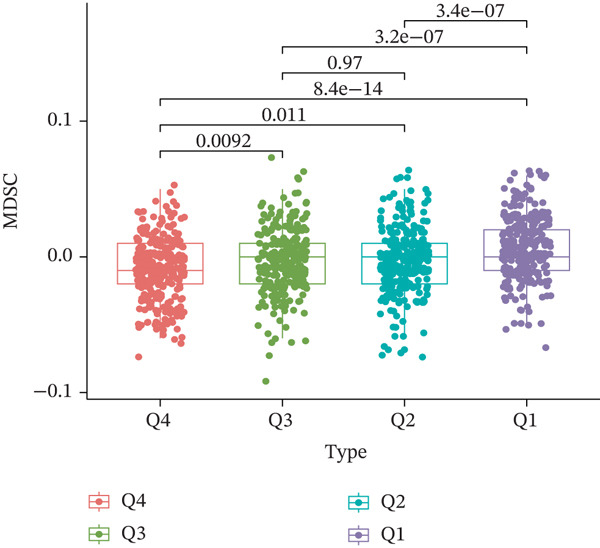
(h)

**Figure:**
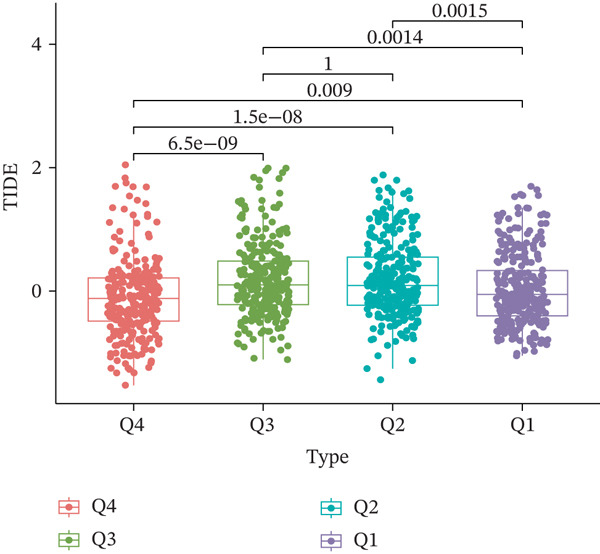
(i)

**Figure:**
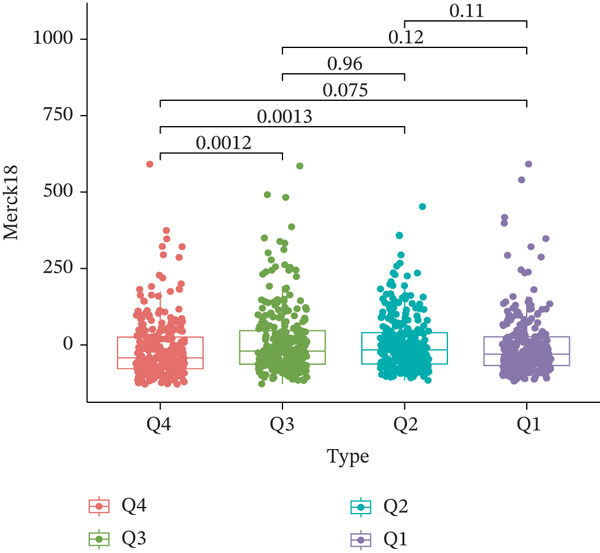
(j)

### 3.4. Correlation Between *HNRNPA3* Expression and Drug Sensitivity Across Multiple Databases

We first integrated outcome data from 25 immunotherapy‐related datasets in the TIDE database with *HNRNPA3* expression levels and performed variance analysis. The results indicated that high *HNRNPA3* expression was associated with increased resistance to immunotherapy (*p* = 0.048) (Figure 5a). These results suggested a potential link between *HNRNPA3* and drug sensitivity. To explore this further, we employed multiple datasets (GDSC1, GDSC2, CTRP, PRISM, and CMAP) and computational algorithms (oncoPredict and XSum) to predict drug sensitivity. Analysis with the oncoPredict package on GDSC1 and GDSC2 datasets revealed strong negative correlations for NPK76‐II‐72‐1 and venetoclax, respectively (Figure 5b,c). Furthermore, by calculating the correlation between drug dose‐response curves (AUC) and *HNRNPA3* expression levels in the CTRP and PRISM databases, we observed notable negative correlations for CD‐437 and talazoparib (Figure 5d,e). These results suggest that higher *HNRNPA3* expression is associated with increased sensitivity to CD‐437 and talazoparib. Through the construction of a network diagram, we visually elucidated the drugs associated with *HNRNPA3* in the GDSC1 and GDSC2 datasets, as well as the biological pathways involved, particularly those related to mitosis, DNA replication, chromatin histone acetylation, cell cycle, and apoptosis regulation (Figure 5f). In the CMAP analysis, exisulind achieved the lowest score, suggesting its potential to reverse the molecular signatures associated with *HNRNPA3* dysregulation. This indicates that exisulind may counteract the oncogenic effects mediated by *HNRNPA3* (Figure 5g).

Figure 5Analysis of *HNRNPA3* expression and drug sensitivity across multiple databases. (a) Association between *HNRNPA3* expression and immunotherapy resistance analyzed using the TIDE database. (b, c) Correlation between *HNRNPA3* expression and drug sensitivity analyzed in the GDSC1 and GDSC2 datasets using the oncoPredict algorithm. (d, e) Correlation between HNRNPA3 expression and drug sensitivity (AUC) analyzed in the CTRP and PRISM databases. (f) Network analysis of *HNRNPA3*‐associated drugs and pathways in the GDSC1 and GDSC2 datasets. (g) Identification of potential therapeutic agents targeting *HNRNPA3* dysregulation using the CMAP database. GDSC, Genomics of Drug Sensitivity in Cancer; CTRP, Cancer Therapeutics Response Portal; PRISM, Profiling Relative Inhibition Simultaneously in Mixtures; CMAP, Connectivity Map; AUC, area under the curve; IC50, half‐maximal inhibitory concentration.

**Figure:**
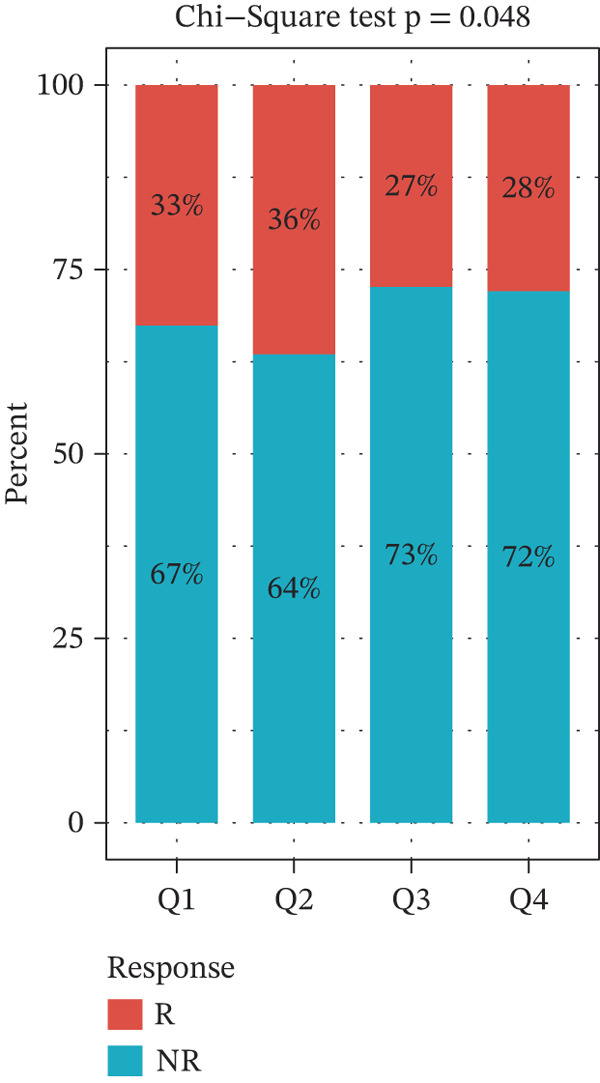
(a)

**Figure:**
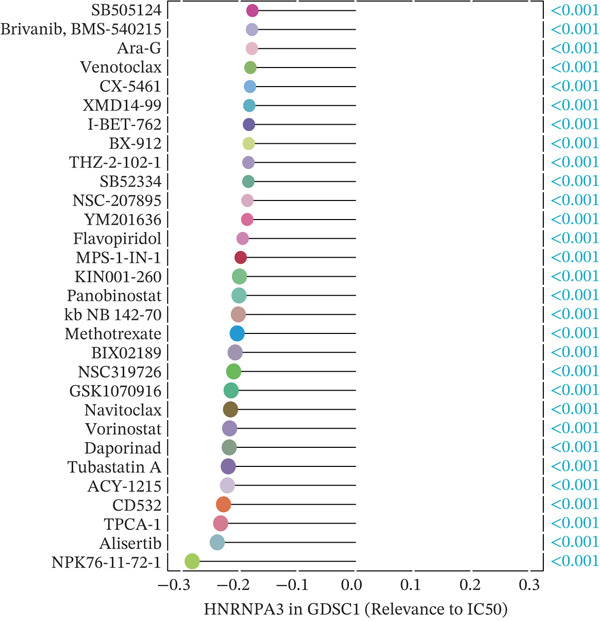
(b)

**Figure:**
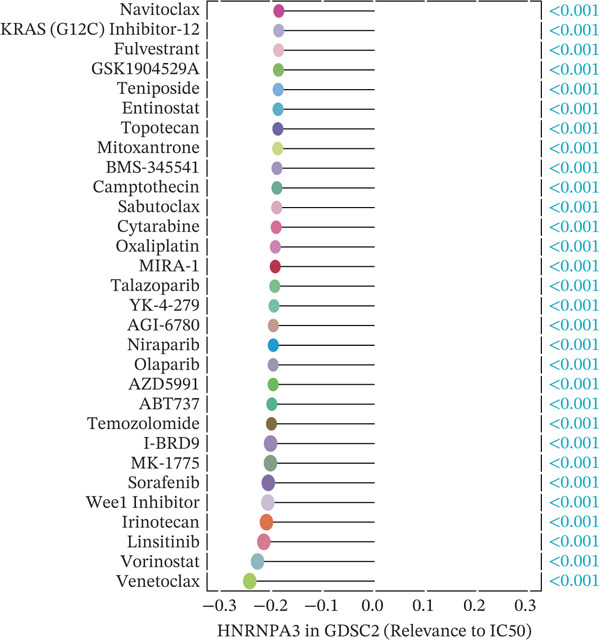
(c)

**Figure:**
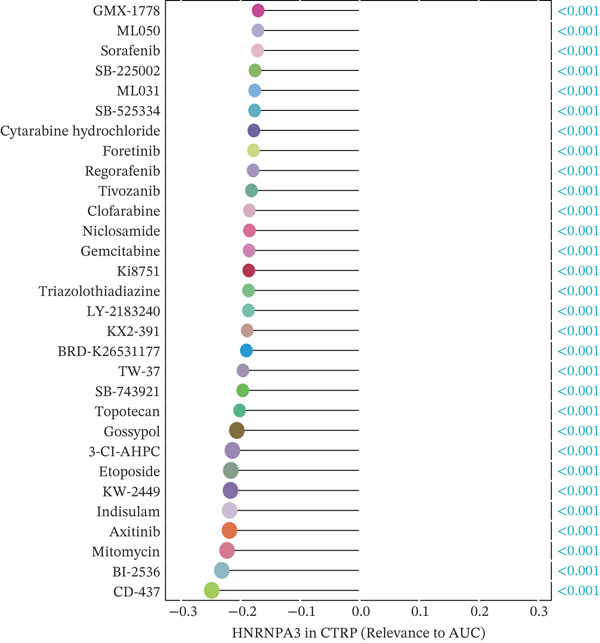
(d)

**Figure:**
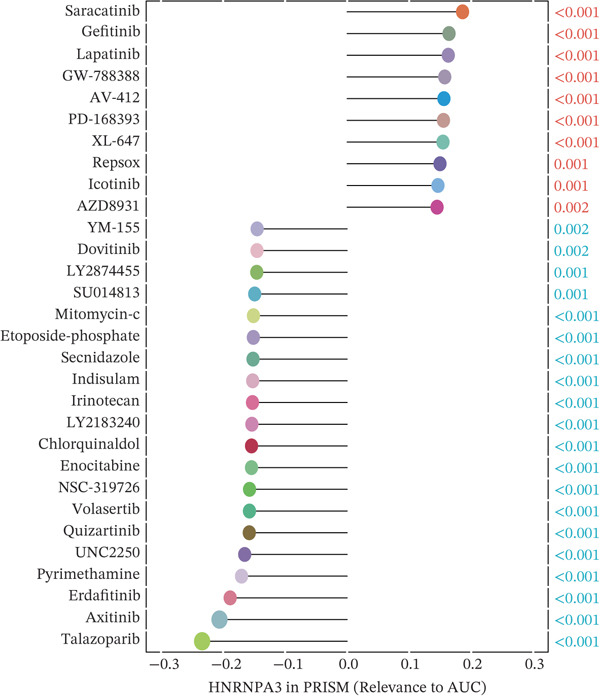
(e)

**Figure:**
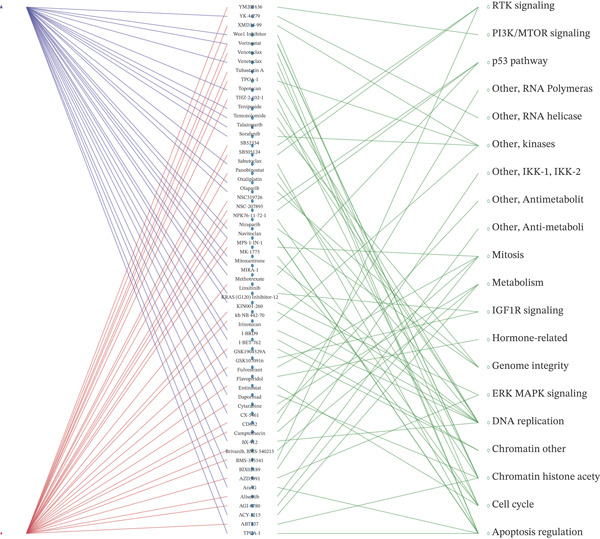
(f)

**Figure:**
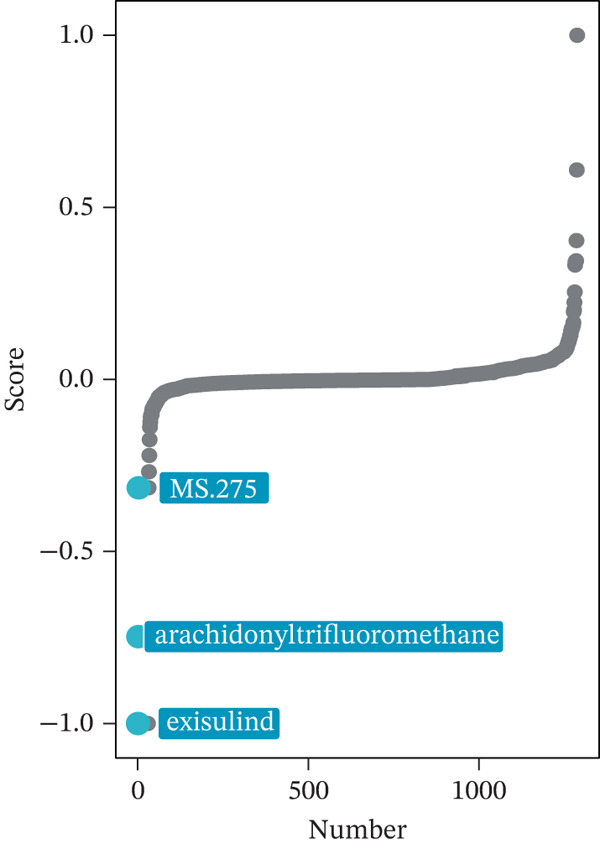
(g)

### 3.5. Mutational Landscape of *HNRNPA3* in BRCA

In the mutational landscape across 33 cancer types, the mutation frequency of *HNRNPA3* was 1%, substantially lower than that of *TP53* (64%) and *PIK3CA* (23%) (Figure 6a). Analysis via the cBioPortal platform identified R52∗ (nonsense mutation) and R103C (missense mutation) as the predominant recurrent mutation sites in *HNRNPA3* (Figure 6b, c). Notably, amplification represented the predominant type of genomic alteration for *HNRNPA3*, rather than point mutations (Figure 6d,e).

Figure 6Genomic alteration landscape of *HNRNPA3* across multiple cancer types. (a) Mutation frequency of *HNRNPA3* compared with key driver genes (*TP53*, *PIK3CA*, *EGFR*, etc.) in 33 TCGA cancer cohorts. (b–c) Recurrent hotspot mutations in *HNRNPA3*, with R52∗ (nonsense) and R103C (missense) as the predominant variants identified via cBioPortal analysis. The protein domain structure (RRM_10 domain spanning residues 378aa) is annotated below the mutation map. (d–e) Distribution of *HNRNPA3* genomic alterations, revealing amplification as the predominant alteration type (up to 0.8% frequency) across cancers, exceeding deep deletions and point mutations. Data were integrated from TCGA Pan‐Cancer Atlas (*n* = 6051 samples) using cBioPortal (http://www.cbioportal.org). Colors denote alteration types: blue, mutation; red, amplification; green, deep deletion.

**Figure:**
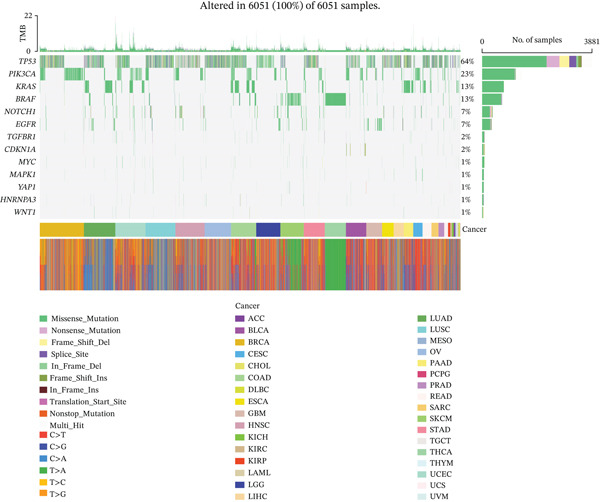
(a)

**Figure:**
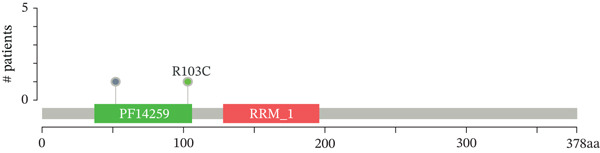
(b)

**Figure:**
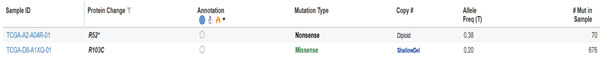
(c)

**Figure:**
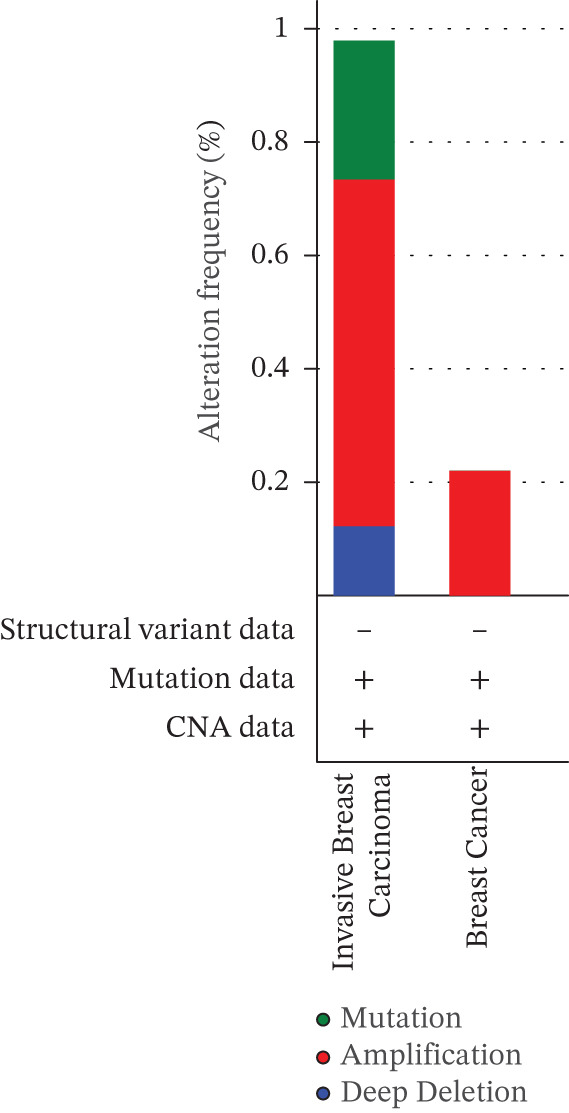
(d)

**Figure:**
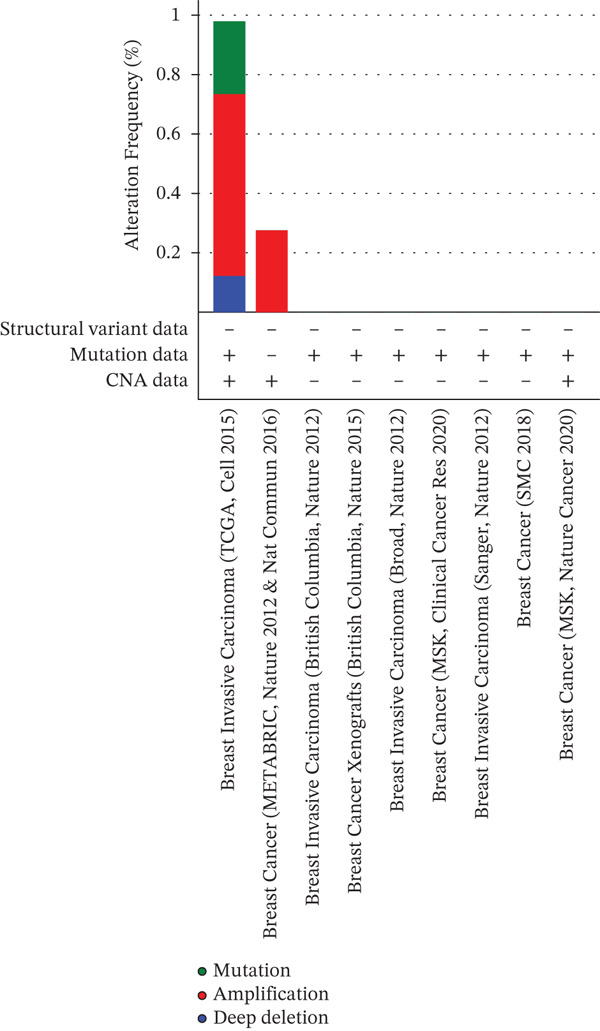
(e)

### 3.6. Expression of *HNRNPA3* in Single‐Cell and Spatial Transcriptomics

In the E‐MTAB‐8107 dataset, high‐density *HNRNPA3* expression predominantly overlapped with T cell and B cell subpopulations (Figures 7a, 7b, and 7c). Similarly, in the GSE176078 dataset, high‐density *HNRNPA3* expression was primarily associated with T cells and malignant cells (Figures 7g, 7h, and 7i). Notably, *HNRNPA3* expression levels were higher in malignant cells compared with immune cells (Figure 7d,j). From a subpopulation perspective, *HNRNPA3* exhibited the highest expression in T proliferating (T prolif) cells across both datasets (Figure 7e,k). To further investigate cellular composition, we categorized all cells into *HNRNPA3*‐positive and *HNRNPA3*‐negative groups based on *HNRNPA3* expression. In the E‐MTAB‐8107 dataset, the *HNRNPA3*‐positive group showed a lower proportion of CD8 T cells but a higher proportion of malignant cells compared to the *HNRNPA3*‐negative group. Similarly, in the GSE176078 dataset, the *HNRNPA3*‐positive group had reduced proportions of CD8 exhausted T cells (CD8Tex) and conventional CD4 T cells (CD4Tconv), whereas the proportion of malignant cells remained higher, consistent with the findings in E‐MTAB‐8107 (Figure 7f,l).

Figure 7Expression patterns of *HNRNPA3* in single‐cell transcriptomics of BRCA. (a) UMAP plot showing cell clusters in the E‐MTAB‐8107 dataset, including 11 cell types: B cells, CD8 T cells, CD8Tex, endothelial cells, fibroblasts, malignant cells, mast cells, monocytes/macrophages (Mono/Macro), myofibroblasts, plasma cells, and T proliferating cells (T prolif). (b–c) UMAP plot and density plot of *HNRNPA3* expression in the UMAP space for the E‐MTAB‐8107 dataset. (d, e) Box plots comparing *HNRNPA3* expression levels among immune cells, malignant cells, and stromal cells (d), and across the 11 cell types (e) in the E‐MTAB‐8107 dataset. (f) Bar plot comparing the proportions of the 11 cell types between *HNRNPA3*‐positive and *HNRNPA3*‐negative groups in the E‐MTAB‐8107 dataset. (g) UMAP plot showing cell clusters in the GSE176078 dataset, including the same 11 cell types. (h–i) UMAP plot and density plot of *HNRNPA3* expression in the UMAP space for the GSE176078 dataset. (j, k) Box plots comparing *HNRNPA3* expression levels among immune cells, malignant cells, and stromal cells (j), and across the 11 cell types (k) in the GSE176078 dataset. (l) Bar plot comparing the proportions of the 11 cell types between *HNRNPA3* ‐positive and *HNRNPA3*‐negative groups in the GSE176078 dataset. UMAP, uniform manifold approximation and projection; CD8Tex, CD8 exhausted T cells; Mono/Macro, monocytes/macrophages; T prolif, T proliferating cells.

**Figure:**
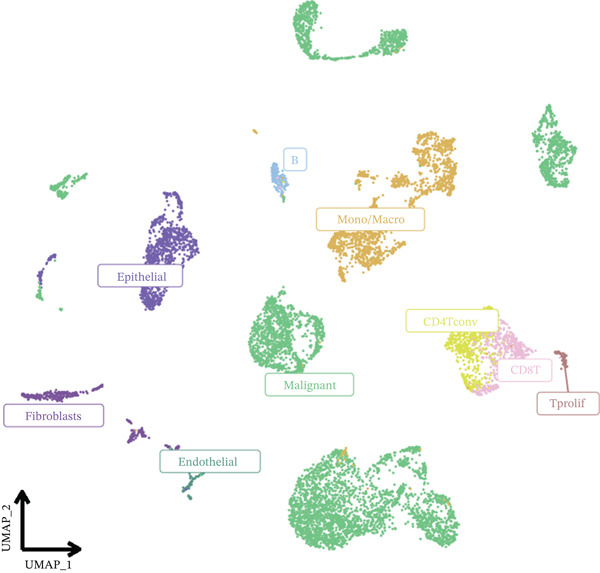
(a)

**Figure:**
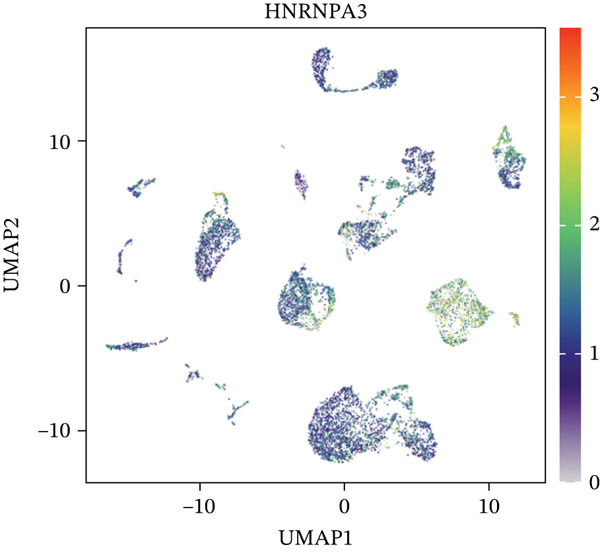
(b)

**Figure:**
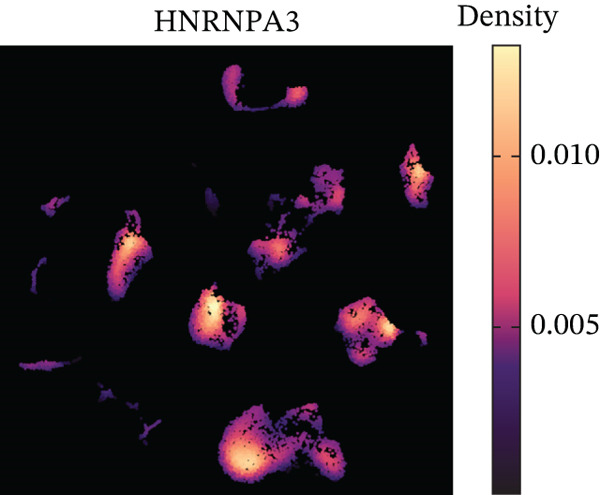
(c)

**Figure:**
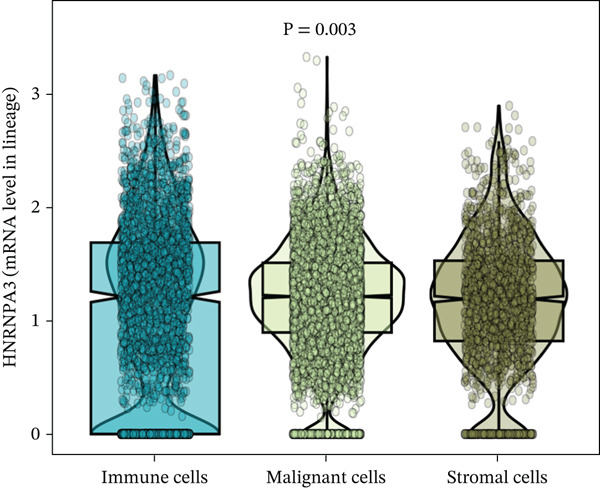
(d)

**Figure:**
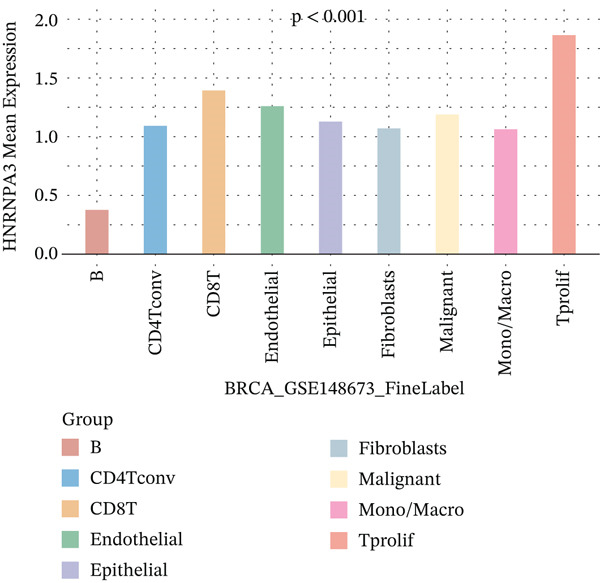
(e)

**Figure:**
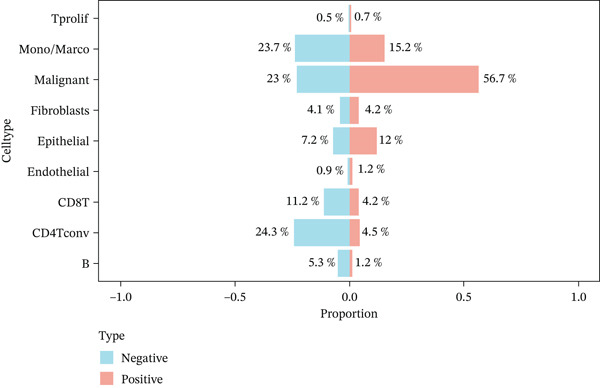
(f)

**Figure:**
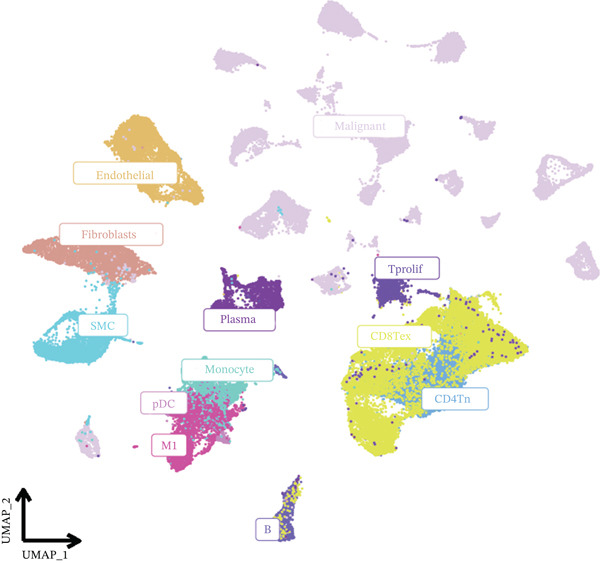
(g)

**Figure:**
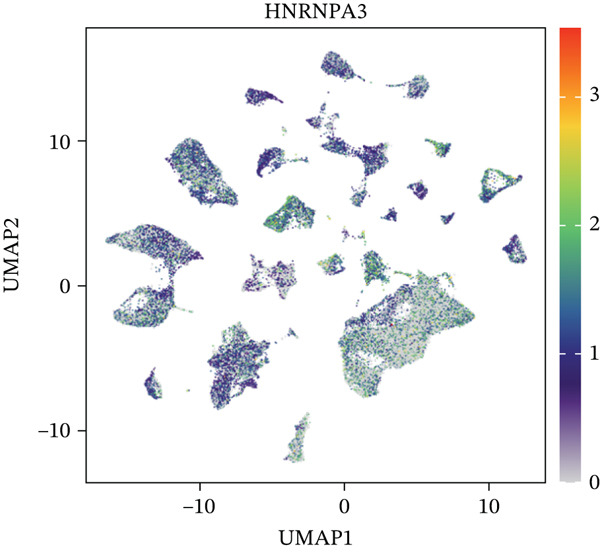
(h)

**Figure:**
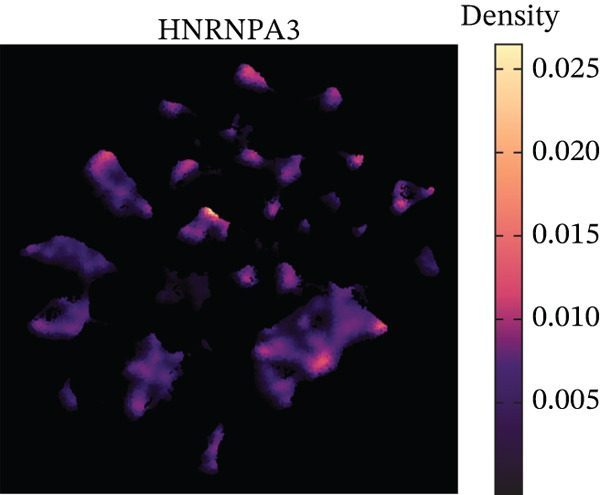
(i)

**Figure:**
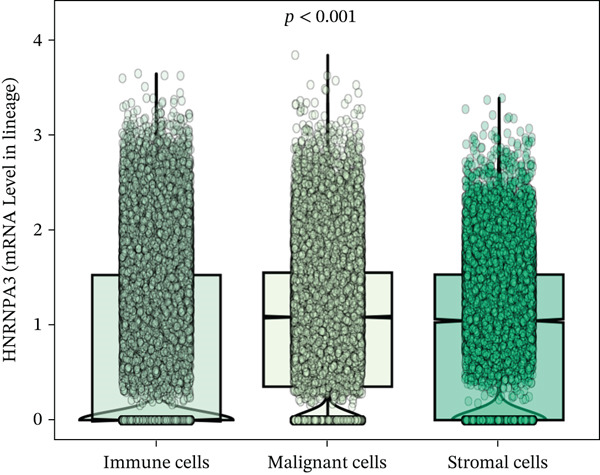
(j)

**Figure:**
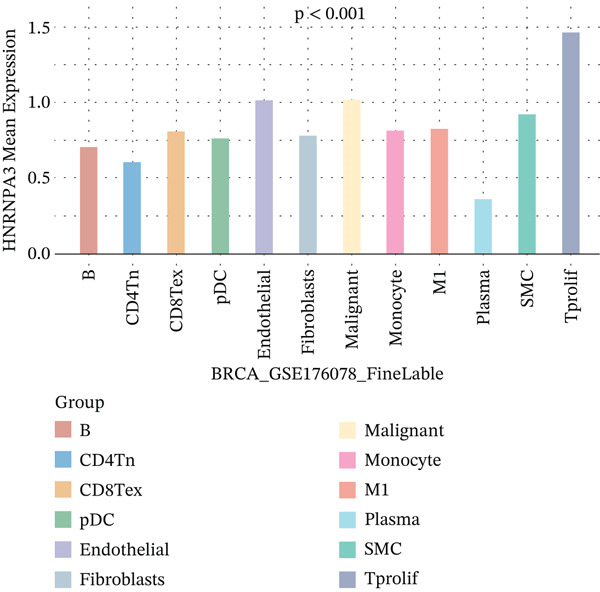
(k)

**Figure:**
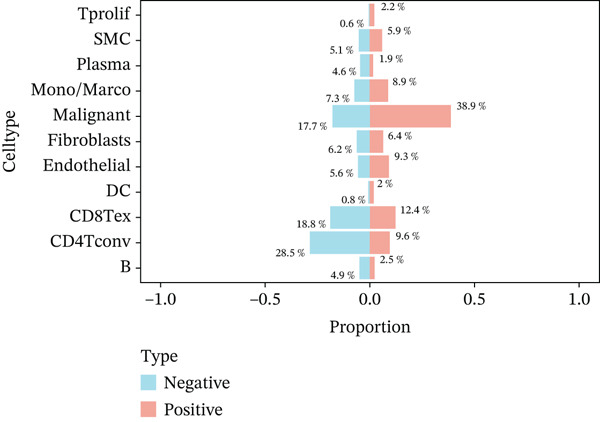
(l)

To further elucidate the expression levels of *HNRNPA3* in breast cancer tissues, we employed spatial transcriptomics to analyze its spatial localization. In the GSM6433588 dataset, *HNRNPA3* exhibited spatial colocalization with tumor cells (Figures 8a, 8b, 8c, and 8d). Notably, *HNRNPA3* expression was significantly higher in the malignant group compared with the mixed and normal groups (*p* < 0.001) (Figure 8e,f). Correlation analysis revealed a strong positive association between *HNRNPA3* and tumor cells, whereas a negative correlation was observed with CD4 cells (Figure 8g). These results indicate that *HNRNPA3* is closely associated with tumor cells in breast cancer, underscoring its potential significance in tumor progression and microenvironment regulation.

Figure 8Spatial expression patterns of *HNRNPA3* in breast cancer tissues. (a) Original pathological tissue section of GSM6433588. (b, c) Spatial localization and relative expression levels of B cells, CD4 T cells, CD8 T cells, dendritic cells (DCs), endothelial cells, fibroblasts, macrophages, plasma cells, and tumor cells in the pathological tissue section. (d) Spatial localization and relative expression levels of *HNRNPA3* in the pathological tissue section. (e) Spatial localization of malignant, mixed, and normal groups in the pathological tissue section. (f) Expression levels of *HNRNPA3* in malignant, mixed, and normal groups. (g) Mantel test correlation heat map between *HNRNPA3* and B cells, CD4 T cells, CD8 T cells, DCs, endothelial cells, fibroblasts, macrophages, plasma cells, and tumor cells.

**Figure:**
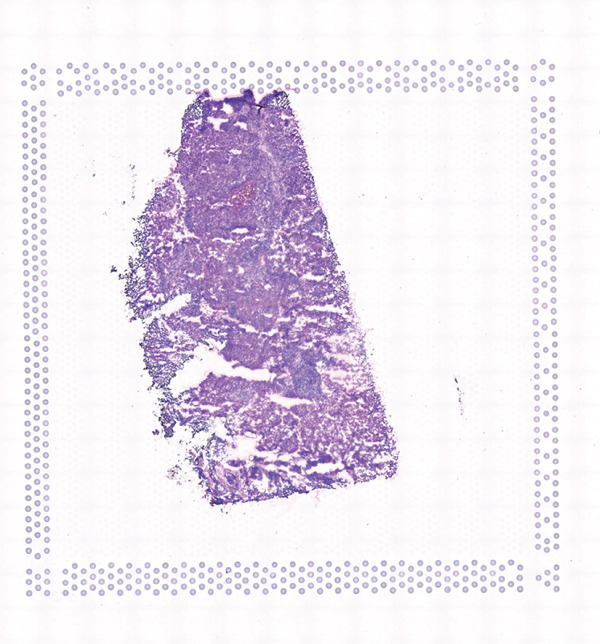
(a)

**Figure:**
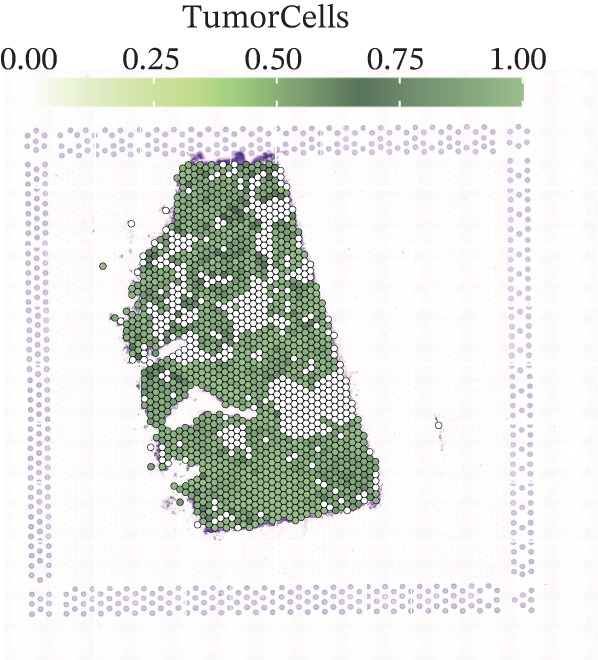
(b)

**Figure:**
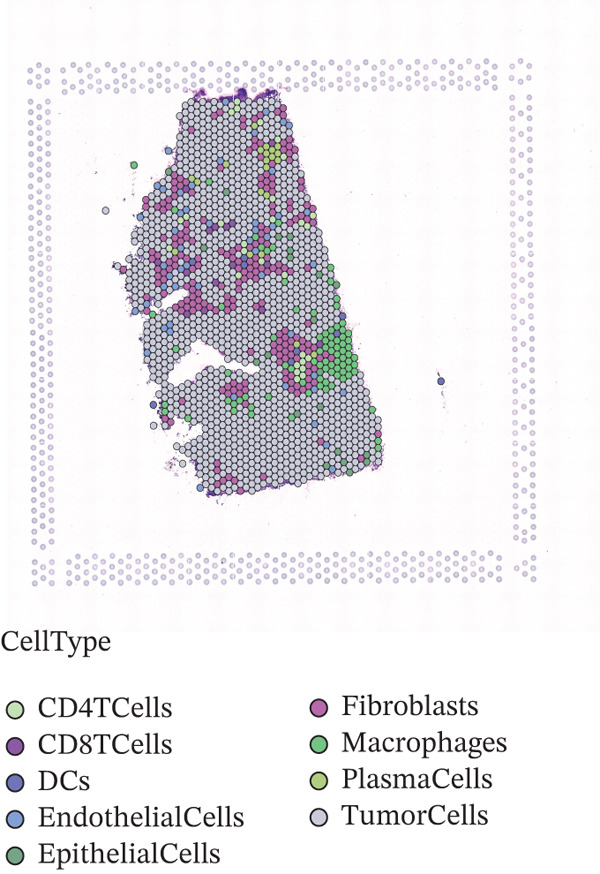
(c)

**Figure:**
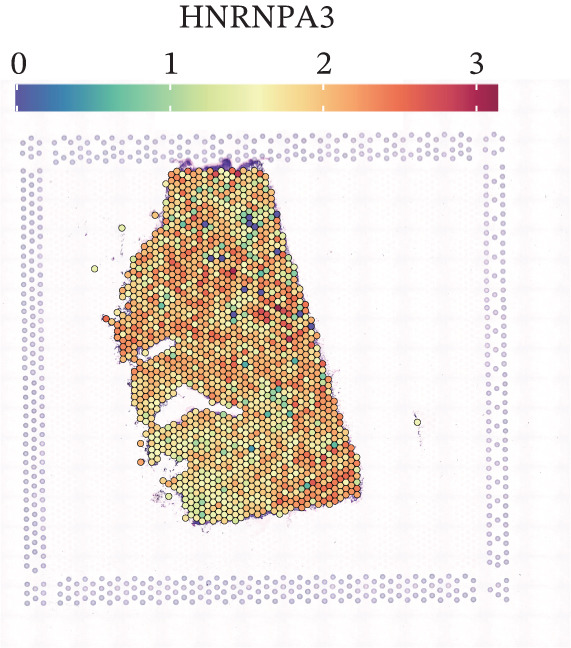
(d)

**Figure:**
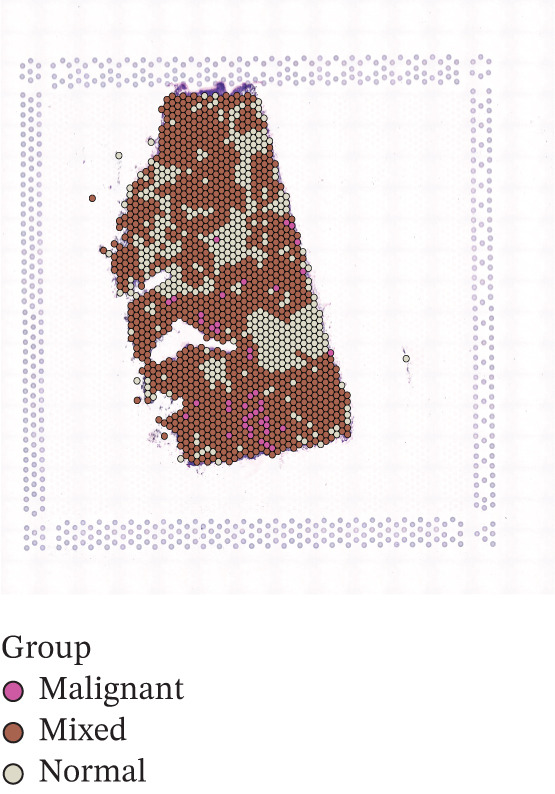
(e)

**Figure:**
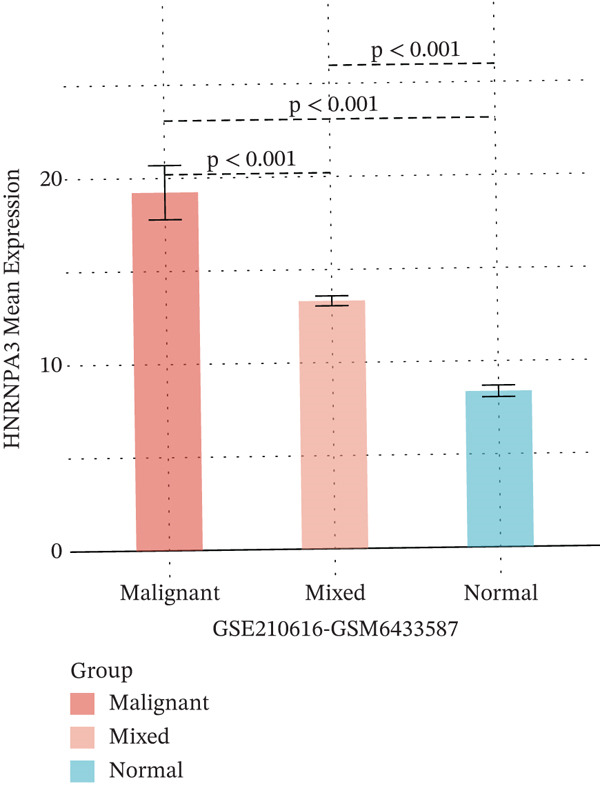
(f)

**Figure:**
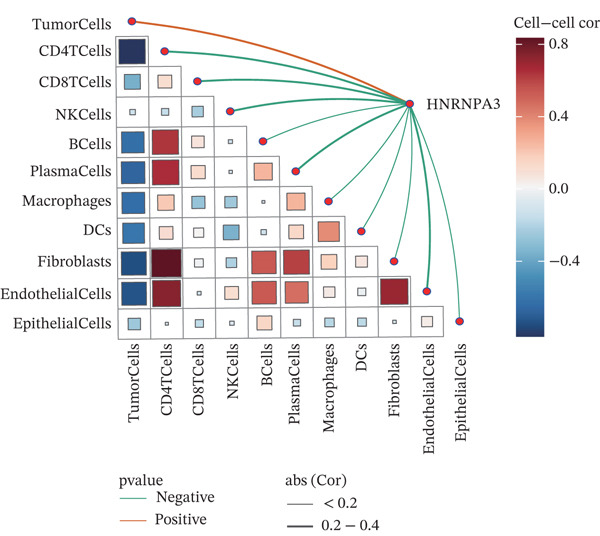
(g)

### 3.7. Validation of *HNRNPA3* in In Vivo and In Vitro Experiments

We first validated the relative mRNA expression levels of *HNRNPA3* in breast cancer cell lines (MDA‐MB‐231, MDA‐MB‐468, MCF7, MDA‐MB‐453, and HCC1806) and the human normal breast cell line Bcap37, as shown in Figure 9a. Subsequently, we overexpressed *HNRNPA3* in MDA‐MB‐231 and MDA‐MB‐453 cells. RT‐PCR analysis confirmed that *HNRNPA3* expression was significantly higher in the overexpression groups (231‐*HNRNPA3* and 453‐*HNRNPA3*) compared with the control groups (pCDH) (*p* < 0.0001) (Figure 9b,c). In a 4‐day cell proliferation assay, the absorbance values of 231‐*HNRNPA3* and 453‐*HNRNPA3* were significantly higher than those of pCDH (*p* < 0.05) (Figure 9d,e). In invasion and migration assays, 231‐*HNRNPA3* and 453‐*HNRNPA3* demonstrated a significantly greater number of invading and migrating cells relative to pCDH (Figure 9f). In the wound healing assay, no significant difference in migration rate was detected between the overexpression and pCDH at Day 1. However, by Day 2, the migration rate was markedly elevated in the overexpression groups compared with pCDH (Figure 9h). To further investigate the role of *HNRNPA3* in breast cancer, we knocked down *HNRNPA3* in MDA‐MB‐453 cells. The results showed that *HNRNPA3* expression in the sh‐*HNRNPA3* group was significantly lower than that in the pCDH group (*p* < 0.01) (Figure 9i). Subsequently, we conducted a tumor formation assay in BALB/c nude mice, which revealed that the tumor volume in the sh‐*HNRNPA3* group was significantly smaller than that in the pCDH (Figure 9j). Furthermore, TUNEL assay analysis of the tumor tissues demonstrated that the positive fluorescence intensity in the sh‐*HNRNPA3* group was significantly higher than that in the control group (Figure 9k).

Figure 9Functional validation of *HNRNPA3* in breast cancer cell lines and in vivo models. (a) Relative mRNA expression levels of *HNRNPA3* in breast cancer cell lines (MDA‐MB‐231, MDA‐MB‐468, MCF7, MDA‐MB‐453, and HCC1806) and the human normal breast cell line Bcap37. (b, c) RT‐PCR analysis of *HNRNPA3* overexpression in MDA‐MB‐231 (231‐*HNRNPA3*) and MDA‐MB‐453 (453‐*HNRNPA3*) cells compared with the pCDH control groups. (d, e) Cell proliferation assay of 231‐*HNRNPA3* and 453‐*HNRNPA3* compared with pCDH. (f) Invasion and migration assays of 231‐*HNRNPA3* relative to pCDH. (g) Invasion and migration assays of 453‐*HNRNPA3* relative to pCDH. (h) Wound healing assay of 231‐*HNRNPA3* and 453‐*HNRNPA3* compared with pCDH. (i) Western blot analysis of *HNRNPA3* knockdown in MDA‐MB‐453 cells (sh‐*HNRNPA3*) compared with the pCDH control group. (j) The tumor formation assay in BALB/c nude mice was performed using MDA‐MB‐453 cells, comparing sh‐*HNRNPA3* and pCDH groups. (k) TUNEL assay analysis of tumor tissues from sh‐*HNRNPA3* and pCDH groups.

**Figure:**
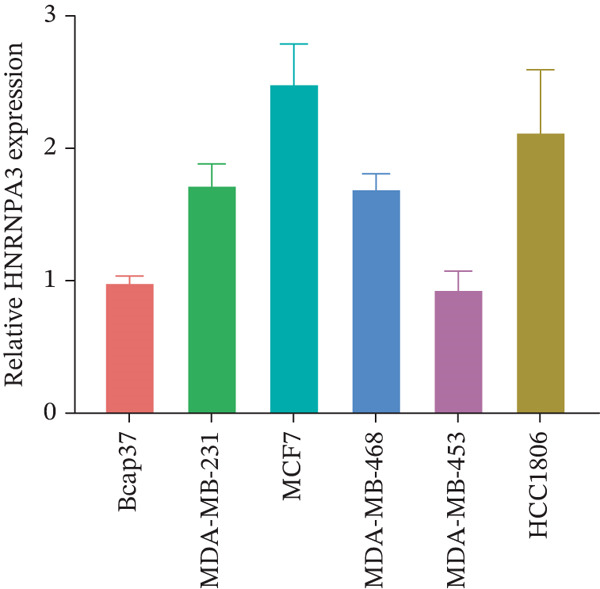
(a)

**Figure:**
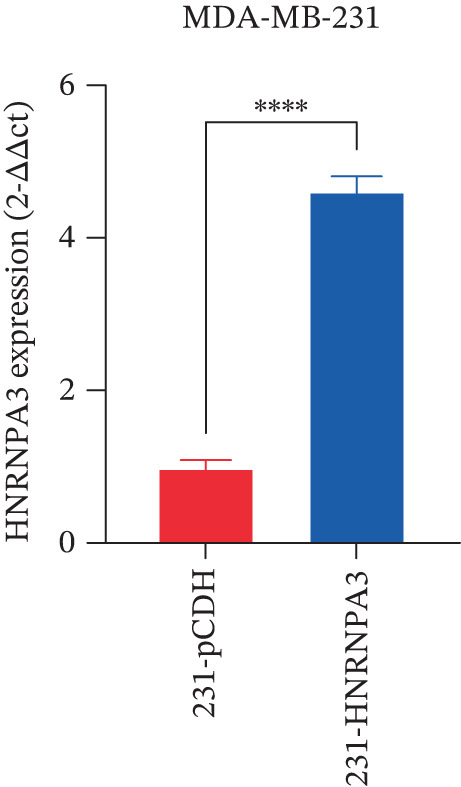
(b)

**Figure:**
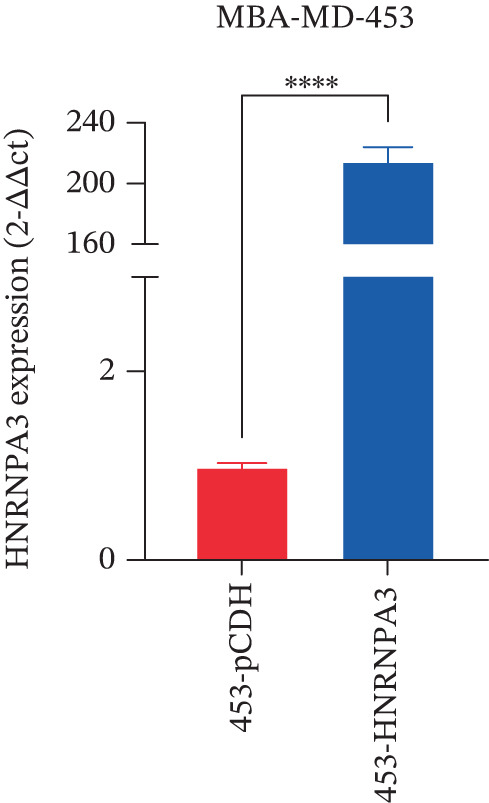
(c)

**Figure:**
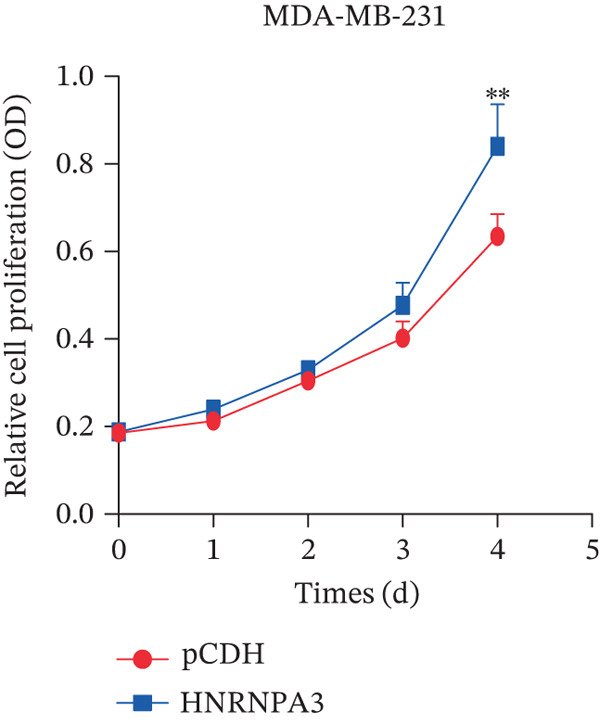
(d)

**Figure:**
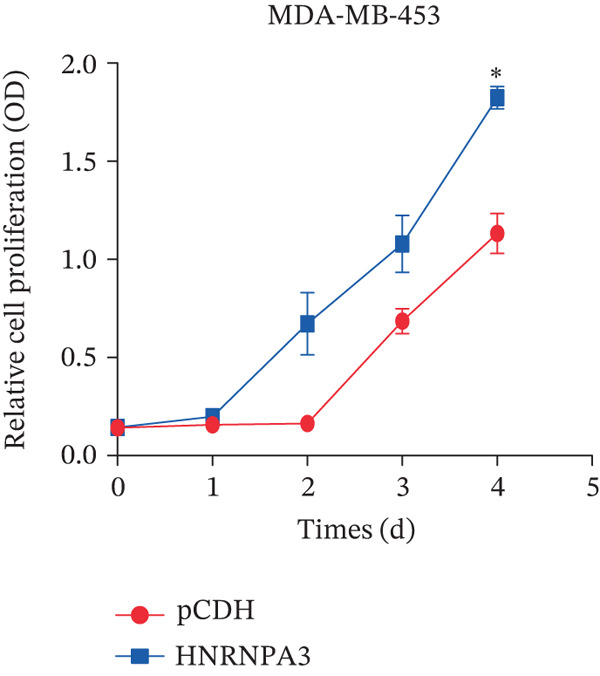
(e)

**Figure:**
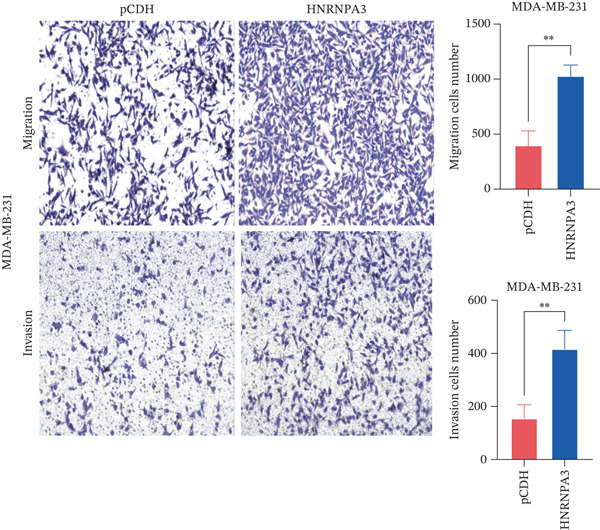
(f)

**Figure:**
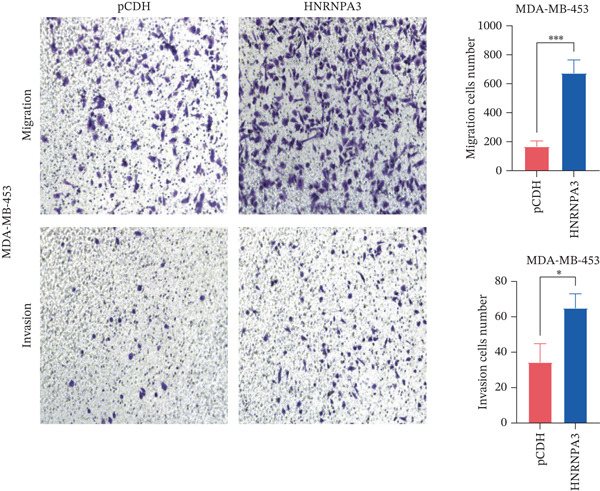
(g)

**Figure:**
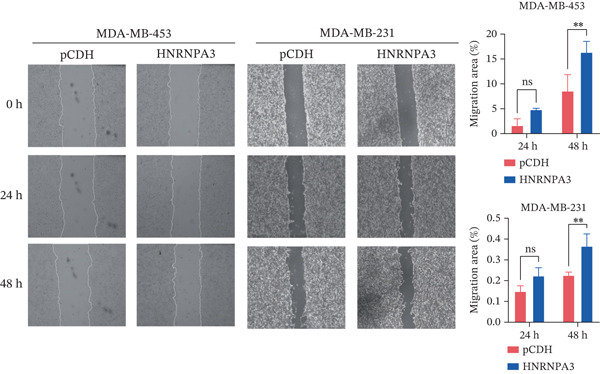
(h)

**Figure:**
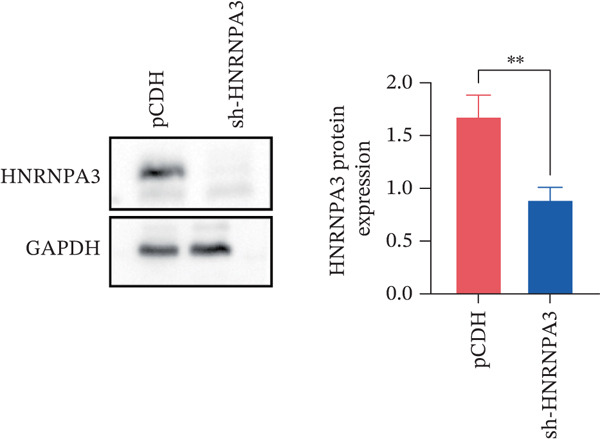
(i)

**Figure:**
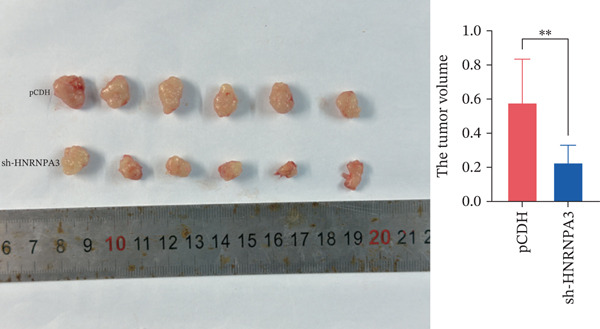
(j)

**Figure:**
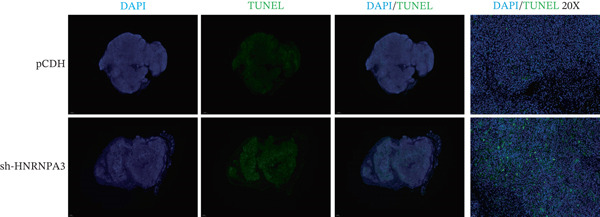
(k)

## 4. Discussion

*HNRNPA3*, a member of the heterogeneous nuclear ribonucleoprotein (hnRNP) family, is known to regulate RNA metabolism, including splicing, transport, and stability [11]. In this study, *HNRNPA3* was found to be significantly overexpressed in high‐grade tumors and metastatic samples, correlating with poor clinical outcomes such as reduced OS and RFS. This aligns with previous studies showing that hnRNPs are frequently dysregulated in cancer and contribute to tumor progression [27, 28]. Furthermore, functional enrichment analysis revealed that *HNRNPA3* is involved in cell cycle regulation and chemokine signaling pathways, which are critical for tumor proliferation and immune evasion, consistent with our in vitro experimental findings [29, 30]. These results collectively suggest that *HNRNPA3* may serve as a key regulator of tumor progression and immune microenvironment dynamics in breast cancer.

Previous studies have shown that members of the HNRNPA/B family, including HNRNPA3, regulate mRNA splicing and stability, thereby influencing the expression of immune‐related genes. For instance, hnRNP L has been shown to regulate the alternative splicing of CD45 mRNA during T cell activation, which may impact T cell function and immune responses (Hui et al. 2003). Similarly, hnRNP I and hnRNP L interact with the 3 ^′^UTR of inducible nitric oxide synthase (iNOS) mRNA, potentially regulating its stability and influencing inflammatory responses and immune signaling [31, 32]. The duality of *HNRNPA3* in sculpting the tumor immune microenvironment—simultaneously fostering immune cell recruitment and enforcing functional suppression—epitomizes a refined immunoediting strategy [33]. Rather than representing a contradiction, this phenotype reflects tumor adaptation: elevated chemokine and immunomodulator signatures associated with *HNRNPA3* likely facilitate initial T‐cell and macrophage infiltration, while concurrent upregulation of immune checkpoints (e.g., PD‐L1) and enrichment of suppressive populations (Tregs, M2 macrophages, MDSCs, and CAFs) establish a functionally restrained niche. Notably, intermediate *HNRNPA3* expression correlates with a heightened yet counterbalanced immune state—marked by elevated cytotoxic indicators (CD8^+^ T cells, and IFN‐*γ*) alongside robust immunosuppressive signals—whereas low expression associates with T‐cell dysfunction, a hallmark of immunotherapy resistance [9]. Mechanistically, as an RNA‐binding protein, *HNRNPA3* may posttranscriptionally coordinate a regulatory network encompassing both chemoattractants and immunosuppressive effectors (e.g., *PD-L1* and *TGFB1*), paralleling mechanisms documented for related hnRNPs [34]. This “recruit‐and‐suppress” paradigm reconciles the disconnect between immune infiltration quantity and effector functionality, providing a coherent explanation for the attenuated immunotherapy response observed in *HNRNPA3*‐high contexts. Future validation of *HNRNPA3′*s direct RNA targets and cell‐type‐specific roles will be pivotal to translate this insight into rational combinatorial strategies—such as *HNRNPA3* inhibition paired with checkpoint blockade—to reprogram the immunosuppressive landscape and restore antitumor immunity.

Drug sensitivity and immunotherapy are complementary therapeutic strategies. In our study, we found that higher *HNRNPA3* expression is associated with elevated CD274 (PD‐L1) levels. CD274, which is frequently overexpressed in various tumor types, binds to PD‐1 on T cells, inhibiting their proliferation and function, thereby enabling tumors to evade immune surveillance. Patients with high CD274 expression are often more responsive to PD‐1/PD‐L1 inhibitors, suggesting that *HNRNPA3* may predict better outcomes in PD‐1/PD‐L1 inhibitor therapy [35]. Additionally, we identified multiple potential small‐molecule targets of *HNRNPA3* that are enriched in key pathways, including mitosis, DNA replication, chromatin histone acetylation, cell cycle regulation, and apoptosis. These findings are consistent with our GSEA results and in vivo experimental data, collectively positioning *HNRNPA3* as a key regulator of immune dynamics and drug sensitivity in breast cancer and providing new insights into its potential as a therapeutic target.

The single‐cell and spatial transcriptomics analyses provided additional insights into the spatial localization and functional roles of *HNRNPA3* in the TME. In both the E‐MTAB‐8107 and GSE176078 datasets, *HNRNPA3* exhibited high expression in malignant cells and T prolif cells, suggesting its role in promoting tumor cell proliferation and immune evasion. The spatial transcriptomics analysis further confirmed that *HNRNPA3* is colocalized with tumor cells and is associated with an immunosuppressive microenvironment, as evidenced by the negative correlation with CD4 cells. These findings are consistent with previous studies showing that hnRNPs can influence immune cell function by regulating the expression of cytokines and chemokines [36]. The integration of single‐cell and spatial transcriptomics data with functional validation experiments highlights the potential of *HNRNPA3* as a therapeutic target in breast cancer. However, our study has several limitations that need to be addressed. For instance, the relationship between *HNRNPA3* and immune regulation requires further validation through in vivo and in vitro experiments. Additionally, the regulatory mechanisms of *HNRNPA3* in apoptosis, including its upstream and downstream pathways, remain to be explored. These questions will be investigated in depth in future studies.

However, several limitations warrant acknowledgment. First, this study primarily leveraged retrospective public datasets (TCGA and GEO), which inherently exhibit intercohort heterogeneity in sample processing, clinical annotation, and demographic variables—potentially influencing result generalizability. Second, computational predictions (immune deconvolution, drug sensitivity scoring, and pathway enrichment) remain algorithm‐dependent; although multiple complementary tools were employed, inherent methodological biases necessitate experimental validation. Third, single‐cell and spatial transcriptomic analyses, though high resolution, are constrained by technical artifacts (e.g., batch effects and transcript dropout) that may obscure subtle cellular dynamics. Finally, although functional assays support bioinformatic inferences, the direct RNA targets of HNRNPA3 and its cell‐type‐specific regulatory mechanisms in the tumor‐immune interface require further dissection via CLIP‐seq, conditional knockout models, and prospective clinical validation. Addressing these limitations will strengthen the translational relevance of HNRNPA3 as a biomarker and therapeutic node in breast cancer precision immunotherapy.

## 5. Conclusion

*HNRNPA3* promotes breast cancer progression and immunotherapy resistance by modulating immune dynamics and drug sensitivity. This study highlights HNRNPA3′s potential as a therapeutic target and provides a theoretical foundation for developing strategies to overcome immunotherapy resistance by targeting HNRNPA3‐mediated pathways.

**Nomenclature**
HNRNPA3: heterogeneous nuclear ribonucleoprotein A3
BRCA: breast invasive carcinoma
TME: tumor microenvironment
scRNA‐seq: single‐cell RNA sequencing
TCGA: The Cancer Genome Atlas
GEO: Gene Expression Omnibus
ROC: receiver operating characteristic
AUC: area under the curve
OS: overall survival
DMFS: distant metastasis‐free survival
PPS: progression‐free survival after progression
RFS: relapse‐free survival
GO: Gene Ontology
KEGG: Kyoto Encyclopedia of Genes and Genomes
NES: Normalized Enrichment Score
GSVA: gene set variation analysis
TIP: tracking tumor immunophenotype
TIDE: tumor immune dysfunction and exclusion
ICB: immune checkpoint blockade
IFNG: interferon gamma
MDSC: myeloid‐derived suppressor cells
CAF: cancer‐associated fibroblasts
GDSC: Genomics of Drug Sensitivity in Cancer
CTRP: Cancer Therapeutics Response Portal
PRISM: Profiling Relative Inhibition Simultaneously in Mixtures
CMAP: Connectivity Map
IC50: half‐maximal inhibitory concentration
XSum: eXtreme Sum
CD8Tex: CD8 exhausted T cells
Mono/Macro: monocytes/macrophages
T prolif: T proliferating cells
DCs: dendritic cells
PD‐L1: programmed death‐ligand 1
PD‐1: programmed cell death protein 1
EMT: epithelial–mesenchymal transition
GSEA: gene set enrichment analysis
FDR: false discovery rate
PCA: principal component analysis
TPM: transcripts per million

## Author Contributions

Lijie Gong and Yang Xu conceived and designed the experiments. Yang Xu and Xufan Cai analyzed the data. Lijie Gong and Weiliang Feng wrote and revised the paper. Lijie Gong, Houquan Tao, and Weihui Guo draw figures. Fanrong Zhang and Jie Ma: funding. Lijie Gong and Yang Xu are co‐first authors and have made equal contributions to this work.

## Funding

This study was supported by Medical Science and Technology Project of Zhejiang Province (10.13039/501100017594, 2022RC016) and the Scientific Research Project of Zhejiang Province Department of Education (Y202044574).

## Ethics Statement

Ethics approval was obtained from Zhejiang Provincial People′s Hospital Ethics Committee No.20241121542102.

## Consent

The authors have nothing to report.

## Conflicts of Interest

The authors declare no conflicts of interest.

## Data Availability

All data generated or analyzed during this study are included in this published article.
